# Transcriptomic analysis of primate placentas and novel rhesus trophoblast cell lines informs investigations of human placentation

**DOI:** 10.1186/s12915-021-01056-7

**Published:** 2021-06-21

**Authors:** Jimi L. Rosenkrantz, Jessica E. Gaffney, Victoria H. J. Roberts, Lucia Carbone, Shawn L. Chavez

**Affiliations:** 1 Department of Molecular and Medical Genetics, Oregon Health and Science University, 3181 S.W. Sam Jackson Park Road, Portland, OR 97239 USA; 2 Division of Reproductive and Developmental Sciences, Oregon National Primate Research Center, 505 NW 185th Avenue, Beaverton, OR 97006 USA; 3 Division of Genetics, Oregon National Primate Research Center, Beaverton, OR 97006 USA; 4 Department of Medicine, Knight Cardiovascular Institute, Oregon Health and Science University, Portland, OR 97239 USA; 5 Department of Medical Informatics and Clinical Epidemiology, Oregon Health and Science University, Portland, OR 97239 USA; 6 Department of Obstetrics and Gynecology, Oregon Health and Science University School of Medicine, Portland, OR 97239 USA; 7 Department of Biomedical Engineering, Oregon Health and Science University School of Medicine, Portland, OR 97239 USA

**Keywords:** Cross-species transcriptomics, Placenta, Non-human primate, Rhesus macaque, Preeclampsia, Pregnancy complications, Trophoblast, Extravillous trophoblast, Reproduction, Evolution

## Abstract

**Background:**

Proper placentation, including trophoblast differentiation and function, is essential for the health and well-being of both the mother and baby throughout pregnancy. Placental abnormalities that occur during the early stages of development are thought to contribute to preeclampsia and other placenta-related pregnancy complications. However, relatively little is known about these stages in humans due to obvious ethical and technical limitations. Rhesus macaques are considered an ideal surrogate for studying human placentation, but the unclear translatability of known human placental markers and lack of accessible rhesus trophoblast cell lines can impede the use of this animal model.

**Results:**

Here, we performed a cross-species transcriptomic comparison of human and rhesus placenta and determined that while the majority of human placental marker genes (HPGs) were similarly expressed, 952 differentially expressed genes (DEGs) were identified between the two species. Functional enrichment analysis of the 447 human-upregulated DEGs, including *ADAM12*, *ERVW-1*, *KISS1*, *LGALS13*, *PAPPA2*, *PGF*, and *SIGLEC6*, revealed over-representation of genes implicated in preeclampsia and other pregnancy disorders. Additionally, to enable in vitro functional studies of early placentation, we generated and thoroughly characterized two highly pure first trimester telomerase (TERT) immortalized rhesus trophoblast cell lines (iRP-D26 and iRP-D28A) that retained crucial features of isolated primary trophoblasts.

**Conclusions:**

Overall, our findings help elucidate the molecular translatability between human and rhesus placenta and reveal notable expression differences in several HPGs and genes implicated in pregnancy complications that should be considered when using the rhesus animal model to study normal and pathological human placentation.

**Supplementary Information:**

The online version contains supplementary material available at 10.1186/s12915-021-01056-7.

## Background

The placenta is the physical link between the mother and fetus as well as a critical site for nutrient and waste exchange during pregnancy. Trophoblasts are the major functional cell type of the placenta and can be divided into three subtypes: (1) proliferative cytotrophoblasts (CTBs), which can differentiate into (2) invasive extravillous trophoblasts (EVTs), or fuse to form (3) multinucleated syncytiotrophoblasts (STBs). Proper trophoblast differentiation and function are essential for normal placentation and fetal development throughout gestation. In humans, abnormal placentation is the primary defect associated with major pregnancy complications, such as preeclampsia, fetal growth restriction, recurrent miscarriage, and still-birth [1]. Investigation of early placentation is particularly important for combating these diseases since many of the associated defects are thought to arise early to mid-gestation. However, the ethical and technical limitations of studying early human development have confined many human placentation investigations to late gestational stages closer to term. Thus, early human placental development, including the origin and cause(s) of the placental abnormalities underlying major pregnancy complications, are poorly understood.

To overcome the limitations of studying early human placentation, numerous human first trimester trophoblast cell lines have been isolated and immortalized using various methods for in vitro investigations [2–4]. Unlike primary trophoblasts, immortalized trophoblast cell lines are readily available, can be grown in culture indefinitely, and are relatively easy to transfect for functional studies. However, recent studies suggest that these cell lines are not necessarily a pure population of trophoblasts and/or have acquired karyotypic and phenotypic aberrations with continued passaging [5–7]. The human choriocarcinoma cell lines, BeWo, JEG-3 and JAR, have also been used to study trophoblast differentiation and syncytialization [8], but these cells are highly malignant and exhibit considerably different transcriptomic profiles than primary trophoblasts [5], questioning whether findings using these lines are truly representative of normal CTBs or EVTs.

While some of the limitations of performing human in vitro and in vivo placental studies are overcome using animal models, most mammalian species poorly recapitulate human placentation. This is due, in large part, to inherent genetic differences and variations in the placental structure, steroidogenic synthesis, and the intracellular signaling pathways mediating lineage-specific trophoblast differentiation amongst mammals [9, 10]. However, non-human primate animal models, particularly rhesus macaques, are genetically similar to humans and share many key features of human placentation. Besides being comparable in placental morphogenesis, the overall structure and nature of both the STB interface layer and intervillous space, as well as endocrine functions and extracellular matrix changes, are similar between rhesus and human placenta [11–14]. Further, there is a strong resemblance between human and rhesus placental endovascular EVT invasion and spiral artery transformation [15], processes known to play a central role in the pathogenesis of several pregnancy complications in humans [16]. Unlike studies relying on human samples, access to high-quality early gestational placental samples and in vivo functional investigations are possible under approved rhesus animal studies. However, rhesus first trimester trophoblast cell lines are still not readily available, which limits rhesus-based placental studies and requires the laborious isolation and use of primary term rhesus trophoblasts for in vitro investigations.

Although the human and rhesus placenta appear to be morphologically and functionally similar, previous studies have revealed some notable differences in the expression level and/or protein-coding potential of well-known human placental markers, including CGA, HLA-G, ERVW-1, and SIGLEC6 [17–20]. Differences in placental invasion have also been noted, as the extent and depth of interstitial EVT invasion is greater in human compared to rhesus placentation [12]. Further, while a few cases of preeclampsia have been documented in rhesus and other non-human primates, this disease predominantly occurs in humans [21–25]. Thus, the identification of the molecular differences between human and rhesus placenta will not only help elucidate the translatability between primate placental studies, but it may also provide valuable insight into the molecular origin of human-specific placental features and pregnancy-related diseases.

Here, we performed a cross-species transcriptomic comparison of human and rhesus placenta to identify differentially expressed genes (DEGs) and determined that even though the majority of human placental marker genes (HPGs) are similarly expressed across the two species, there are gene expression differences that likely underlie the distinct features of human placentation. Additionally, we generated and thoroughly characterized two highly pure TERT-immortalized rhesus trophoblast cell lines for in vitro functional studies that retained features of primary rhesus trophoblasts. Overall, this work provides a comprehensive list of genes differentially expressed between human and rhesus placenta that enhances the translatability of primate placental investigations and helps delineate the molecular differences underlying human susceptibility to preeclampsia and other pregnancy-related diseases. It also offers previously unavailable first trimester immortalized rhesus trophoblast cell lines for further functional investigation and understanding of early primate placentation.

## Results

### Identification of genes differentially expressed between human and rhesus placenta

**Fig. 1:**
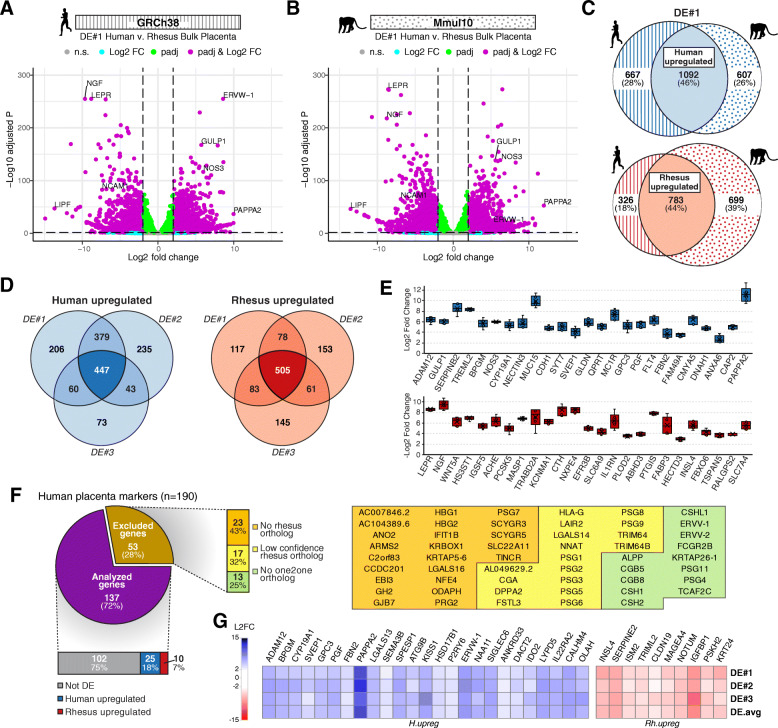
Cross-species transcriptional comparison of human and rhesus bulk placental tissue. Volcano plots showing gene expression fold differences between human (*n*=*6*) and rhesus (*n*=4) term placental tissue from DE#1, using data mapped to **a** the human genome and **b** the rhesus genome. Dashed lines denote DE significance (padj<0.05) and fold change (|L2FC|>2) thresholds; genes passing padj threshold (green), L2FC threshold (cyan), both (magenta), or none (gray). **c** Venn-diagram depicting intersection of DEGs identified using data mapped to human genome (stripes) and rhesus genome (spotted) to identify intermediate human-upregulated (light blue) and rhesus-upregulated (light red) genes sets. **d** Venn-diagram depicting the intersection of the results from the three DE analyses, to identify the final set of 447 human-upregulated genes (blue), and 505 rhesus-upregulated genes (red) (Additional file 3). **e** Top 25 most significant differentially expressed (ranked by mean padj) human-upregulated (blue) and rhesus-upregulated genes (red); box plots depict average Log_*2*_ fold change of each gene from the three DE analyses. **f** Differential expression results of HPGs. (Left) Proportion of placental marker genes analyzed (purple) or excluded from (brown) DE analysis. Analyzed genes are further classified as either not differentially expressed (not DE) (gray), human-upregulated (blue), or rhesus-upregulated (red). (Right) ENSEMBL-classification of HPGs excluded from DE analysis; “no rhesus ortholog” (orange), “low confidence rhesus ortholog” (yellow), or “no one2one rhesus ortholog” (green). **g** Heatmap of differentially expressed HPGs; human-upregulated genes (L2FC>2) are shown in blue and rhesus-upregulated genes (L2FC<2) are shown in red

Despite the structural and functional similarities between human and rhesus placentas, differences in the level and route of invasion, as well as the increased propensity to develop pregnancy-related diseases in humans, suggests that molecular differences exist across primate species. To characterize such differences, we compared gene expression levels between human and rhesus macaque (*Macaque mulatta*) placentas using a combination of newly generated and publicly available RNA-seq data from bulk third trimester placenta samples [26]. The presence of EVTs in rhesus placental samples at this gestational time-period was confirmed via immunohistochemical (IHC) staining of the pan-trophoblast marker, KRT7 (Additional file 1: Fig. S1). In order to make the RNA-seq gene expression data comparable across species (Additional file 2), we limited our comparison to human protein-coding genes with ENSEMBL-defined “high-confidence” “one2one” rhesus orthologs and DEGs were identified by intersecting the results of two complementary differential expression (DE) analyses (Additional file 1: Fig. S2A). First, RNA-seq data from both species were mapped to the human reference genome (GRCh38) (Fig. 1a) and DEGs were identified based on the human gene annotations (DE-GRCh38). Second, RNA-seq data from both species were mapped to the rhesus reference genome (Mmul10) and DEGs were identified based on the rhesus gene annotations (DE-Mmul10) (Fig. 1b). A gene was considered differentially expressed only if it was identified as significantly (padj<0.05) upregulated or downregulated (|L2FC|>2) by both analyses (Fig. 1c). Thus, DEGs called due to mappability issues instead of true expression differences would not be called in the reciprocal analysis and excluded from the final set of DEGs. To avoid potential batch effects, we repeated the DE analysis using three independent human placenta RNA-seq datasets [27, 28] (Additional file 1: Fig. S1B, C). This final set of DEGs consisted only of genes determined to be significantly upregulated or downregulated by all three DE analyses and resulted in a total of 952 DEGs, including 447 human-upregulated and 505 rhesus-upregulated genes (Fig. 1d; Additional file 3). Quantitative reverse transcription PCR (qRT-PCR) was used to validate eight of the DEGs (Additional file 1: Fig. S3A, B). The 25 most significant human and rhesus upregulated DEGs are shown in Fig. 1e and highlights the upregulation of several well-known placental markers in human including, *ADAM12*, *SERPINB2*, *BPGM*, *CYP19A1*, *SVEP1*, *GPC3*, *PGF*, *FBN2*, and *PAPPA2*. Collectively, these results provide a comprehensive list of gene expression differences between human and rhesus placenta and show that not all established human placental markers are expressed equivalently in the two species.

### Inclusion of mouse placenta confirms the overall molecular similarity in placentation between primates

To validate the primary DE analysis and provide an unbiased assessment of the molecular similarity between human, rhesus, and mouse [29] placenta, an orthologous cross-species transcriptomic comparison was performed that relied exclusively on RNA-seq data mapped to each respective species genome. We used an approach similar to the one recently described by Sun et al. [30], in which transcripts per million (TPM) normalized gene expression values were calculated for each sample (Additional files 4, 5, 6), then filtered to include only genes with one-to-one orthologs across all three species (human, rhesus, and mouse) for cross-species comparison. Consistent with Sun et al, hierarchical clustering of TPM-normalized expression data showed that rhesus placental samples were more closely-related to human than mouse (Additional file 1: Fig. S4A, B; Additional file 7). A total of 1787 DEGs, including 879 human-upregulated and 814 rhesus-upregulated DEGs, were identified between human and rhesus placenta using TPM-normalized expression data (Additional file 8). Of the 952 DEGs identified in our primary DE analysis, 58% (*n*=554) were also identified by the TPM-based analysis (Additional file 1: Fig. S4C). It should be noted that ~8% (73/952) of DEGs in our primary analysis were excluded from the novel TPM-based approach due to a lack of ENSEMBL-defined one-to-one human-to-mouse ortholog, including preeclampsia associated genes (*ERVW-1*, *KISS1*, *LGALS13*, *SIGLEC6*) and HPGs (*GPC3*, *INSL4*, *MAGEA4*, *NAA11*, *OLAH*, *PSKH2)* (Additional file 1: Fig. S4D, E). Thus, while inclusion of mouse in the TPM-based transcriptomic comparison allowed for an unbiased assessment of molecular similarities between human and rhesus, it also minimized the number of orthologous genes that could reliably be compared, thereby excluding several key DEGs identified by our primary analysis. Nonetheless, the results of the TPM-based comparative analysis confirms the overall molecular similarity between human and rhesus placenta, as well as the stringency and confidence of our primary DE approach and the final DEG set reported.

### Cross-species comparison reveals enrichment of HPGs implicated in pregnancy-related disorders

To elucidate the translatability of human placental markers between human and rhesus, a set of previously defined HPGs [31] was examined for differential expression between the two species. Out of the 190 HPGs, ~72% (*n*=137/190) were included in the primary DE analysis, while 28% (*n*=53/190) were excluded due to nonexistent (43%), low confidence (32%), or a lack of “one2one” (25%) ENSEMBL-defined rhesus ortholog (Fig. 1f). In certain cases, ENSEMBL-defined orthology conflicted with orthologous genes described by previous studies. For instance, the *GH2* gene is defined as having no ENSEMBL rhesus ortholog despite the well-described placentally expressed *GH/CS* locus in the rhesus genome that is highly similar to human [32–34]. Further, both *HLA-G* and *CGA* are defined as having only “low-confidence” rhesus orthologs, opposed to previous studies describing highly similar orthologous genes in the rhesus genome [17, 18]. Of the 137 HPGs included in the analysis, the vast majority (~75%; *n*=102/137) showed similar expression levels between human and rhesus placenta. The remaining ~25% of HPGs (*n*=35/137) were identified as differentially expressed between the two species, with ~18% (*n*=25/137) upregulated in human and ~7% (*n*=10/137) upregulated in rhesus placenta (Fig. 1f; Additional file 9). Notably, several HPGs associated with invasive EVTs (*ADAM12*, *PAPPA2*, *PGF*) [35–37], and pregnancy complications such as preterm birth and preeclampsia (*ADAM12*, *HSD17B1*, *KISS1*, *LGALS13*, *PAPPA2*, *SIGLEC6*, *ERVW-1*) [38–44], were found to be upregulated in human compared to rhesus placenta (Fig. 1g). Over-representation analysis (ORA) demonstrated that genes associated with pregnancy disorders including, “preeclampsia” (padj=6.73E-04), “HELLP syndrome” (padj=1.48E-01), “Gestational trophoblastic tumor” (padj=1.52E-01), and “Eclampsia” (padj=4.43E-01) were indeed upregulated in human compared to rhesus placenta (Additional file 1: Fig. S5; Additional file 10). Overall, these results provide a comprehensive list of differentially expressed HPGs between human and rhesus placenta that should be considered when studying certain aspects of placentation in rhesus, particularly those that are associated with EVT invasion, preeclampsia, or other placenta-related diseases.

### DEGs detected between human and rhesus placenta largely reflect species-specific changes

While both the human and rhesus RNA-seq data used for our DE analysis was obtained from placenta samples collected from third trimester cesarean sections without labor, we note that the publicly available rhesus data was generated from a slightly earlier (~80%) gestational age (GA) than the term human placental samples. Therefore, it is possible that some of the gene expression differences observed between the human and rhesus samples may be the result of GA rather than species-specific changes. However, examination of a set of previously defined “GA-specific” genes expressed in primate placentas [45] revealed that only a single GA-specific gene (*BAALC*) was identified as differentially expressed in our analysis. In addition, closer examination of the rhesus placenta RNA-seq data showed little to no expression of Y-linked genes in any of the samples, suggesting an unequal distribution of male and female samples may have influenced the cross-species comparison. To determine whether any of the DEGs identified were due to sex-specific rather than species-specific differences, we compiled a set of “sex-differentially expressed” (SDE) genes via DE analysis of known male (*n*=6) and female (*n*=5) human placentas. A total of 11 significant SDE genes were identified (Additional file 11), five of which overlapped with our human-upregulated DEGs (*ZFY*, *RPS4Y1*, *KDM5D*, *DDX3Y*, *CCK*). Therefore, sex-specific changes accounted for only ~0.53% (5/952) of the DEGs in the cross-species analysis of human and rhesus placentas. These results indicate that the DEGs identified in our study largely reflect species-specific changes rather than GA-related or sex-specific differences.

### Establishment of TERT-immortalized rhesus placenta and skin fibroblast cell lines

**Fig. 2:**
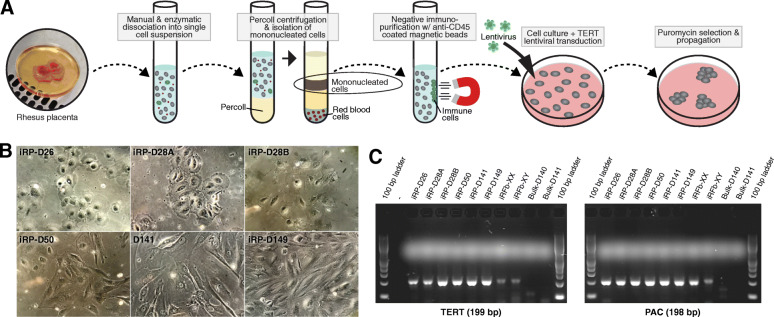
Establishment of TERT immortalized rhesus placental and skin fibroblast cell lines. **a** Schematic of primary trophoblast cell isolation from rhesus placental tissue and TERT immortalization. **b** Phase contrast microphotographs of immortalized placental cell lines (*n*=6). **c** Confirmation of TERT immortalization via RT-PCR detection of *TERT* and *PAC* gene expression

Because the placenta is a heterogeneous organ comprised of many cell types in addition to trophoblasts, such as immune, stromal, and vascular cells [46], we next sought to isolate primary trophoblasts from bulk rhesus placentas for immortalization and characterization, including a comparison of gene expression. While previous studies have successfully isolated and cultured primary trophoblasts from first and third trimester rhesus placenta [47–50], as well as generated rhesus blastocyst- and placenta-derived trophoblast stem cells [51, 52], no rhesus immortalized trophoblast cell lines currently exist for in vitro investigations. Using the strategy described in Fig. 2a, we isolated primary trophoblast cells from rhesus placental tissues collected at gestational day 26 (~6 weeks human pregnancy), day 28 (~7 weeks human pregnancy), day 50 (~12 weeks human pregnancy), day 141 (~34 weeks human pregnancy), and day 149 (~35 weeks human pregnancy). After depletion of contaminating immune cells using immunopurification, the cells were cultured for 24 h before transduction with lentivirus containing *TERT* and puromycin resistance (*PAC*) genes for antibiotic selection. First and third trimester primary cell isolation methods resulted in ~98% and ~69% cytokeratin (KRT7)-positive trophoblast cells, respectively (Additional file 1: Fig. S6). A total of six immortalized rhesus placental (iRP) cell lines were generated, including four from first trimester (iRP-D26, iRP-D28A, iRP-D28B, iRP-D50) and two from third trimester (iRP-D140, iRP-D141) rhesus placentas. Male and female rhesus primary skin fibroblasts were also used to establish two immortalized rhesus fibroblast (iRFb) cell lines, iRFb-XY and iRFb-XX, as controls. Cultures of iRP-D26 and iRP-D28A contained purely polygonal epithelial-like cells, while the other cell lines (iRP-D28B, iRP-D50, iRP-D140, iRP-D141) appeared heterogeneous with a mix of large flattened and elongated fibroblast-like cells (Fig. 2b). Expression of the lentiviral-transduced genes, *TERT* and *PAC*, was confirmed in each of the cell lines via RT-PCR (Fig. 2c). These results suggested that the lentiviral-based TERT-transduction strategy was quite robust, with 100% (*n*=8/8) of attempts resulting in the generation of a cell line with stable *TERT* expression. Moreover, both the iRP-D26 and iRP-D28A have been cultured beyond 30 passages, further supporting successful TERT-immortalization of these cell lines.

### Assessment of genomic integrity in TERT-immortalized rhesus cell lines

**Fig. 3:**
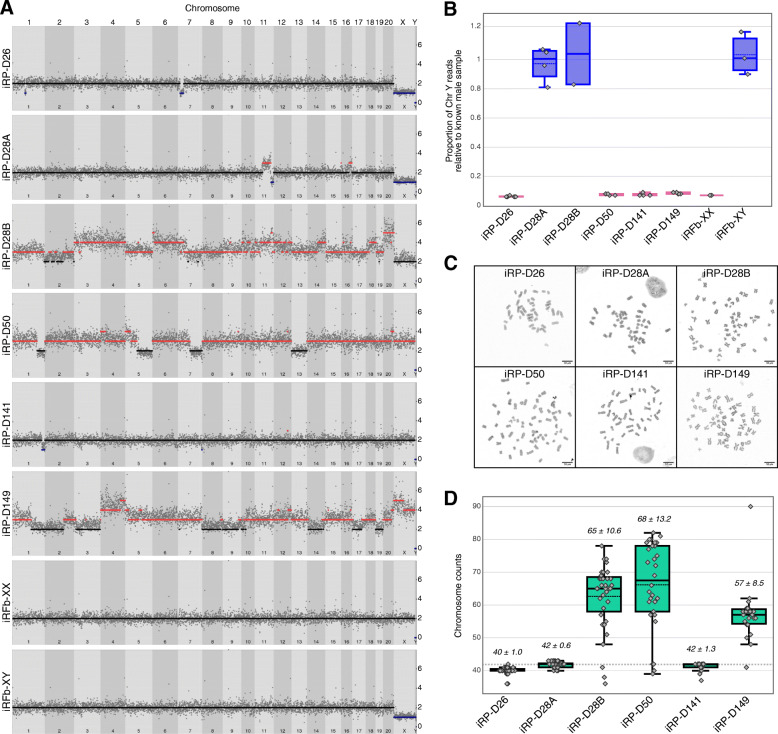
Assessment of genomic integrity in immortalized placental cell lines. **a** Manhattan plot of whole genome CNVs organized by chromosome; copy number gains (red), copy number losses (blue) show the presence of CNV in most of the placental cell lines but not in the fibroblasts used for comparison. **b** Box plots depicting proportion of Chr Y mapped reads normalized to a known male sample, iRFb-XY; cell lines were identified as male if mean > 0.5 (blue), or as female if mean < 0.5 (pink). **c** Representative metaphase spreads (*n*=20); 10uM scale bar. **d** Box plots of chromosome count results; median ± standard deviation values included above each plot

Cell lines are known to develop genetic abnormalities during immortalization and/or prolonged cell culture, such as aneuploidies, copy number variations (CNVs), and chromosomal fusions. This is particularly true for cell lines derived by Simian Virus 40 (SV40) or a similar transformation approach as has been shown for first trimester human trophoblast cell lines [53, 54], but it can also occur in TERT-immortalized human trophoblasts over time [7]. In addition, primary trophoblasts normally undergo cell fusion (syncytialization), which can complicate nuclear assessment. Therefore, CNVs and whole chromosome counts were examined in the cell lines using low-input DNA-seq and metaphase spreads, respectively. Approximately ten cells from each TERT-immortalized cell line were manually transferred into a single tube and prepared for DNA-seq as previously described [55]. Normal diploid copy numbers were observed for all autosomes in iRP-D26, iRP-D28A, and iRP-D141, although sub-chromosomal losses of Chr1 and Chr7 were observed in iRP-D26, sub-chromosomal gains of Chr11 and Chr16 in iRP-D28A, and a small sub-chromosomal loss of Chr1 in iRP-D141 (Fig. 3a). In contrast, numerous whole and sub-chromosomal CNVs were identified in the other iRP cell lines, iRP-D28B, iRP-D50, and iRP-D149. As expected, CNV analysis of the female rhesus fibroblasts (iRFb-XX) showed normal diploid copy numbers for all 21 rhesus chromosomes, while the male fibroblasts (iRFb-XY) exhibited the expected ChrX “loss” and detection of ChrY. Comparison to the male iRFb-XY control revealed a single copy of ChrX without the detection of ChrY in iRP-D26, highlighting the loss of a whole sex chromosome (Fig. 3b). Metaphase spreads of iRP-D26 cells confirmed the loss of one to two whole chromosomes, supporting the DNA-seq results, and suggesting the existence of chromosome fusion in cells with only 40 chromosomes (Fig. 3c). Examination of metaphase spreads from iRP-D28B, iRP-D50, and iRP-D149 further demonstrated a heterogenous mix of predominantly polyploid cells, containing between three (triploid) and four (tetraploid) sets of chromosomes (Fig. 3d). Overall, these results suggest that TERT-immortalization can be used to establish normal diploid rhesus placental cell lines, but we expect that these cells cell could accumulate chromosomal abnormalities with continued passaging as has been shown for human TERT-immortalized trophoblast cell lines [7, 56].

### iRP-D26 and iRP-D28A represent two highly pure immortalized rhesus trophoblast cell lines

**Fig. 4:**
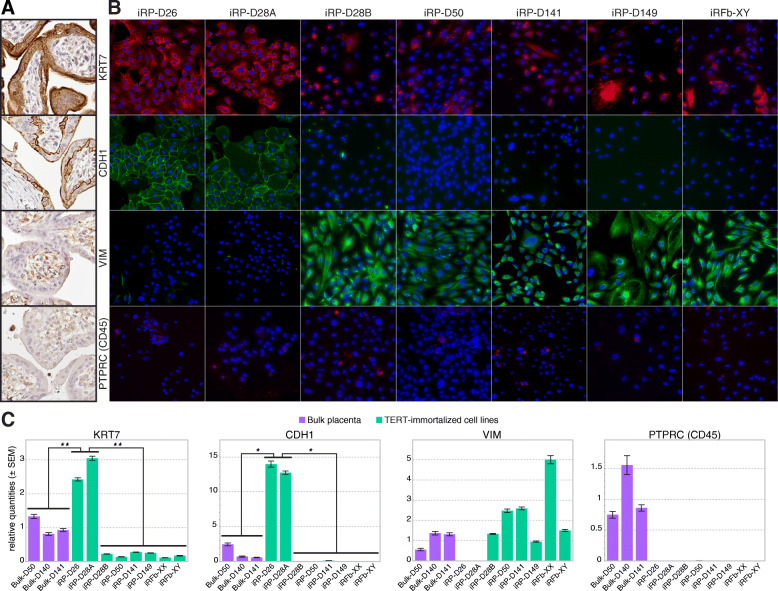
iRP-D26 and iRP-D28A represent two highly pure rhesus immortalized trophoblast cell lines. **a** IHC staining of gestational day 50 rhesus placental tissue for mononuclear trophoblast (KRT7 and CDH1), stromal (VIM), and immune cell (PTPRC) markers (DAB, brown); hematoxylin nuclear counter stain (blue). **b** IF staining of immortalized cell lines for KRT7 (red), CDH1 (green), VIM (green), and PTPRC (red); DAPI nuclear counterstain (blue); results show that iRP-D26 and iRP-D28A cells express known mononuclear trophoblast markers, KRT7 and CDH1 (*n*=3). **c** Bar graphs of qRT-PCR expression results; bulk rhesus placental samples (purple, *n*=3), iRP cell lines (green, *n*=8). Statistical significance was determined using two-sided unpaired t test with alpha of 0.05 (*p<0.05, **p<0.01)

In order to identify the placental cell lines containing a pure population of trophoblast cells, we analyzed each line for expression of highly conserved trophoblast and non-trophoblast cell markers [57], including KRT7, a pan-trophoblast cell marker; CDH1, a mononuclear trophoblast cell marker; VIM, non-trophoblast stromal marker; and PTPRC (CD45), a pan-leukocyte marker. Antibodies for these markers were first validated in rhesus placental tissues using IHC staining and the observed expression patterns were consistent with known patterns in the human placenta (Fig. 4a). Immunofluorescent (IF) staining using the same antibodies showed robust staining of KRT7 and CDH1 trophoblast markers, and the absence of VIM and CD45 staining in both iRP-D26 and iRP-D28A, indicating the enrichment of trophoblasts and the absence of mesenchymal and immune cells within these cell lines, respectively. In contrast, the remaining iRP cell lines (iRP-D28B, iRP-D50, iRP-D141, and iRP-D149) were largely contaminated with VIM-positive mesenchymal cells (Fig. 4b). These findings were consistent with qRT-PCR expression analysis, which showed significant enrichment of KRT7 and CDH1 expression in iRP-D26 and iRP-D28A compared to the other cell lines or bulk rhesus placental tissue (Fig. 4c). Additionally, VIM and PTPRC (CD45) expression was not detected by qRT-PCR in iRP-D26 and iRP-D28A, but were highly expressed in all other cell lines analyzed. Thus, despite careful trophoblast isolation procedures, only 50% (*n*=2/4) of the attempts with first trimester placentas and 0% (*n*=2/2) with third trimester placentas resulted in highly pure immortalized trophoblast cell lines. This indicated that contamination of non-trophoblast stromal cells occurred in ~67% (*n*=4/6) of the primary trophoblast isolations and that only the iRP-D26 and iRP-D28A cell lines should be carried forward for further characterization.

### Transcriptomic comparison of immortalized and primary rhesus trophoblast cells

**Fig. 5:**
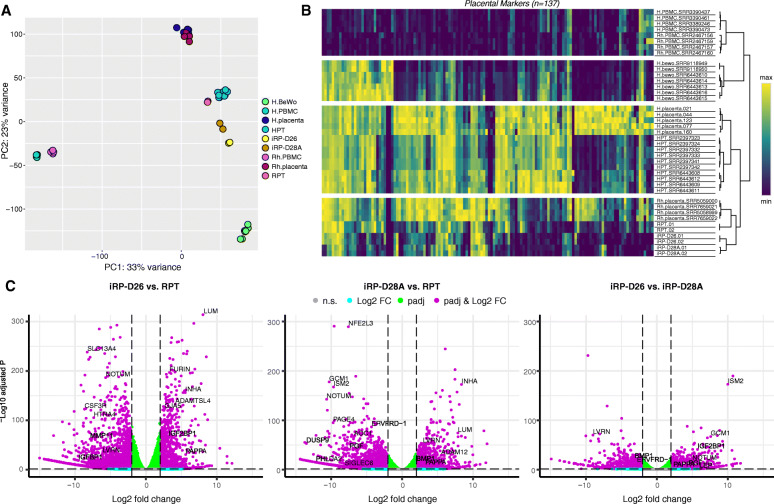
Transcriptomic comparison of immortalized and primary rhesus trophoblast cells. **a** PCA plot of RNA-seq gene expression from human (*n*=6) and rhesus (*n*=4) bulk placentas, human (*n*=4) and rhesus (*n*=4) PBMCs, human primary trophoblasts (*n*=10), BeWo cells (*n*=7), and the iRP-D26 (*n*=2) and iRP-D28A (*n*=2) cell lines. **b** Heatmap of HPG expression results. Color scale depicts minimum (purple) and maximum (yellow) one plus Log_2_ normalized expression values compared across all human and rhesus samples. **c** Volcano plots of DE analysis results; positive L2FC values represent genes upregulated in iRP-D26 compared to RPT (left; Additional file 12), iRP-D28A compared to RPT (middle; Additional file 13), and iRP-D26 compared to iRP-D28A (right)

To characterize gene expression levels in the immortalized trophoblast cell lines and compare them to first trimester rhesus primary trophoblast (RPT) cells, RNA-seq was performed on two replicates each of iRP-D26, iRP-D28A, and freshly isolated first trimester RPTs. RPT trophoblast purity was confirmed via KRT7 staining and showed ~98% KRT7+ trophoblast cells (Additional file 1: Fig. S6). As a reference, publicly available human and rhesus peripheral blood mononuclear cells (PBMC) [58, 59], human primary trophoblasts (HPT) [60, 61], BeWo [61, 62], and rhesus bulk placenta [26] RNA-seq datasets were also included in the assessment. Principle component analysis (PCA) based on the expression of all analyzed genes (*n*=15,787) revealed that a majority of the sample variance was due to tissue-type differences, separating the PBMC samples from the placental/trophoblast samples (Fig. 5a). Bulk human and rhesus placenta samples clustered closely together, further supporting the overall molecular similarity between these two closely related species. Both iRP-D26 and iRP-D28A clustered with the primary trophoblast samples, indicating that our newly generated immortalized trophoblast cell lines were most similar to freshly isolated RPT cells. In contrast, the BeWo samples formed a distinct cluster away from the other trophoblast samples, confirming the major transcriptomic differences between primary trophoblasts and this widely used choriocarcinoma model. Despite broad transcriptomic similarities across the human and rhesus placenta/trophoblast samples, distinct HPG expression was also observed between the two species. Hierarchical clustering of the samples based on this expression showed clustering of placenta/trophoblasts by species, with iRP-D26, iRP-D28A, RPT, and bulk rhesus placenta samples forming a distinct branch and HPT and bulk human placenta forming another (Fig. 5b). Thus, with the exception of HPGs, transcriptomic profiles were largely shared between our newly generated immortalized trophoblast cell lines and freshly isolated RPT cells, demonstrating the suitability of these lines for in vitro primate placental studies.

By performing a DE analysis between (1) iRP-D26 vs. RPT and (2) iRP-D28A versus RPT, we identified DEGs and specific pathways in the immortalized trophoblasts. Out of the total 21,575 protein-coding rhesus genes examined, 5884 DEGs (padj<0.05 & |L2FC|>|1|) were found between iRP-D26 and RPT, with ~39% (*n*=2,290) upregulated and ~61% (*n*=3,594) downregulated in iRP-D26 (Fig. 5c; Additional file 12). Further, a total of 6017 DEGs were identified between iRP-D28A and RPT, with ~43% (*n*=2592) upregulated and ~57% (*n*=3425) downregulated in iRP-D28A (Fig. 5c; Additional file 13). ORA demonstrated that genes associated with herpes simplex virus 1 (HSV1) infection were upregulated in both iRP-D26 and iRP-D28A compared to RPTs. Since HSV1 infection is known to increase TERT activity [63], upregulation of HSV1-associated genes in both immortalized trophoblast cell lines is likely a result of TERT-immortalization induced expression changes. In addition, “EVT-enriched,” “STB-enriched,” and “CTB-enriched” gene sets derived from a previous single-cell RNA-seq analysis of first trimester human placenta [64] were included in the ORA between the cell lines and RPTs. While genes associated with human EVTs and extracellular matrix organization were upregulated in both iRP-D26 and iRP-D28A, genes associated with human STBs, CTBs, and the immune system were downregulated in both immortalized trophoblast cell lines relative to RPTs (Additional file 1: Fig. S5; Additional files 14, 15). These results suggest that the immortalized trophoblast lines are most similar to the previously described human EVT trophoblast cell population [64] and that the RPT samples likely contained a more heterogenous population of trophoblast subtypes than the immortalized trophoblast cell lines. Thus, downregulation of immune response-related genes in the cell lines may be due to the absence of specific trophoblast subtypes (CTB and/or STB), or the presence of cytokine-stimulated trophoblasts within the freshly isolated RPT samples.

### Functional characterization of iRP-D26 and iRP-D28A immortalized trophoblast cell lines

**Fig. 6:**
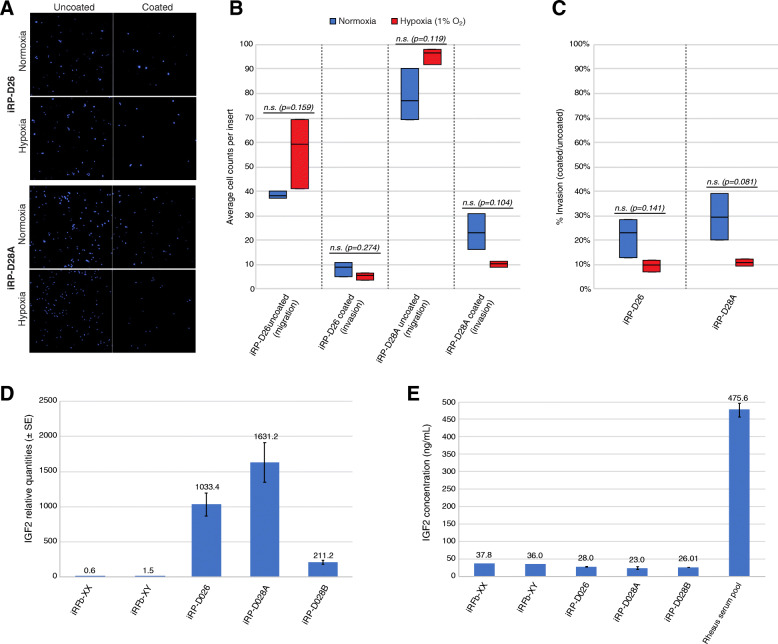
Functional characterization of iRP-D26 and iRP-D28A. **a** Representative micrographs of iRP-D26 and iRP-D28A trans-well inserts after migration and Matrigel invasion assays. Nuclei were counterstained with DAPI (blue). **b** Box plot of average cell counts (*n*=5) from uncoated (migration, *n*=3) and Matrigel-coated (invasion, *n*=3) inserts under normoxic (blue) and hypoxic (red) conditions. A two-sided unpaired t test with alpha of 0.05 was used to determine significance. **c** Box plot of percent invasion (ratio of invasive cells relative to migratory cells) determined for each of the cell lines under normoxic (blue) and hypoxic (red) conditions. **d** Bar chart of IGF2 qRT-PCR expression levels of iRFb (*n*=2) and iRP samples (*n*=4). **e** Bar chart of IGF2 protein secretion levels for iRFb (*n*=2), iRP (*n*=3), and the rhesus serum pool (*n*=4); error bars depict standard error (SE)

In order to test whether iRP-D26 and iRP-D28A behaved more like CTBs or EVTs, the fusogenic potential of the cell lines was assessed by treating them with forskolin, an activator of adenylate cyclase and known inducer of fusion and STB formation in BeWo human choriocarcinoma [65] and trophoblast stem cells [66]. Unlike RPTs and/or forskolin treatment of BeWo cells, neither iRP-D26 or iRP-D28A showed upregulation of the key fusogenic/STB genes, ERVFRD-1 and ERVW-1 (Additional file 1: Fig. S7A-D). Rather, increased RNA expression of several STB markers (SDC1, BMP1, GCM1) was observed in forskolin treated iRP-D26 cultures, as well as regions of reduced CDH1 plasma membrane staining. However, closer examination of these regions by confocal imaging did reveal a faint intact membrane surrounding most nuclei, and only a low-level of SDC1 (a STB marker) expression [67] (Additional file 1: Fig. S7E-F). Taken together, it appeared that the iRP-D26 cell line was sensitive to forskolin treatment, but did not undergo complete STB formation. Additionally, monkey CG (mCG) secretion could not be detected in the culture media from either the iRP-D26 or iRP-D28A cell line (Additional file 1: Fig. S7G). Since mCG is primarily secreted by the syncytiotrophoblast and reduced and/or disrupted CDH1 staining is associated with both invasive cancerous and trophoblast cells [68, 69], this further indicated that the cell lines might be more EVT-like in origin. Indeed, both iRP-D26 and iRP-D28A showed upregulation of several genes known to facilitate human EVT and/or tumor cell invasion (Additional files 12, 13), including *SDC2*, *TIMP3*, *MMP14*, and *ADAM12* [70–73]. Thus, to evaluate whether the immortalized trophoblast cell lines were capable of invasion, trans-well migration and Matrigel extracellular matrix invasion assays were performed. When grown on uncoated transmembrane inserts (*n*=3) for 48 h, both the iRP-D26 and iRP-D28A cell lines exhibited migration to the bottom side of the insert, indicating that the cell lines possessed migratory capabilities (Fig. 6a, b). From these cells, 22% of the iRP-D26 and 30% of the iRP-D28A line were also able to invade to the other side of Matrigel-coated inserts (*n*=3) after 48 h of culture (Fig. 6c). However, despite previous studies showing an increased level of EVT invasion under hypoxic conditions [74, 75], no significant differences in invasion were identified when the assays were performed under hypoxic (1% O_2_) compared to normoxic conditions. This further supports the idea that even though they are prevalent throughout pregnancy (Additional file 1: Fig. S1), EVTs in rhesus placentas appear to be less invasive than their human counterparts. Nonetheless, the EVT-like characteristics of both iRP-D26 and iRP-D28A were confirmed by high expression levels of *IGF2* using qRT-PCR (Fig. 6d), which is most abundantly expressed by EVTs in both human and rhesus [76, 77]. Although high levels of *IGF2* mRNA was detected, however, iRP-D26 and iRP-D28A did not exhibit substantial IGF2 protein secretion (Fig. 6e) that is known to promote EVT migration [78, 79]. Thus, culture of these cell lines with IGF2 supplemented media and/or co-culture with IGF2 secreting cells may enhance their migration and invasive abilities in subsequent studies.

## Discussion

The wide variety of placental morphologies and physiologies that exist among mammals makes it difficult to adequately model human placentation and placental pathologies in other species [80–82]. However, many distinctive features of human placentation are reportedly conserved in rhesus monkeys [11–15]. Despite this conservation, no study has been conducted to comprehensively assess the molecular similarities and potential differences between human and rhesus placental tissues. Comparative analyses of human and closely related species are beginning to identify specific genetic and molecular changes that seem to account, in part, for specific aspects of human evolution, including human diseases [83]. Thus, identification of the molecular differences between human and rhesus placenta is not only needed to elucidate the translatability between human and rhesus placental studies, but it may also provide valuable insight into the molecular origin of human placental diseases, such as preeclampsia. Here, we performed a cross-species transcriptomic comparison of human and rhesus placental tissue in order to identify molecular differences and ultimately elucidate the translatability between human and rhesus placental investigations. Further, to increase the accessibility of rhesus in vitro placental studies, we generated and thoroughly characterized two highly pure TERT-immortalized rhesus trophoblast cell lines that retained features of primary rhesus trophoblasts.

Our DE analysis of human and rhesus placental tissue revealed that while the majority of HPGs were similarly represented, certain genes were differentially expressed between the two species. Specifically, genes associated with preeclampsia and several well-known invasive EVT markers were upregulated in human compared to rhesus placenta. These results are consistent with previously reported differences between human and rhesus placentation, such as the increased extent and depth of interstitial EVT invasion [11, 12, 84] and heightened risk of preeclampsia in humans [85]. Even though preeclampsia is thought to originate in the first trimester, many of the molecular and/or cellular processes that occur during first trimester can also occur at lower levels or in a smaller number of cells in third trimester placentas, including EVT differentiation and invasion. Additionally, third trimester placental abnormalities are well documented in cases of preeclampsia [86], and thus, examination of third trimester placental tissues may still provide insight into the genes associated with preeclampsia as shown here. While this study was not designed to provide mechanistic insight into preeclampsia, our findings are not unexpected considering that preeclampsia occurs frequently in humans and rarely in rhesus and other primates. Comparison of first trimester human and rhesus placental transcriptomes would likely identify more significant differences and/or numerous additional human-upregulated genes related to preeclampsia. However, it is difficult to simultaneously analyze first trimester placentas and allow the pregnancy to continue unless chorionic villous samples are collected as part of a prenatal screening. Nonetheless, our results provide novel insight into the molecular differences underlying human and rhesus placentation and an evolutionary perspective of how preeclampsia and other pregnancy-related diseases may have arisen during human development.

Despite the existence of several prior placental transcriptomic comparisons across distantly related species [87, 88], the majority of the DEGs identified here have not been previously reported. Our study was different in that we not only examined highly conserved orthologous genes, but were also able to compare and identify differential expression of recently evolved primate placental genes such as *LGALS13* [89]. In particular, we show that *LGALS13* is significantly upregulated in human compared to rhesus placenta. This gene encodes galectin-13, which interacts with glycoproteins and glycolipids to facilitate the expansion of uterine arteries and veins during pregnancy in an endothelial cell-dependent manner via the eNOS and prostaglandin signaling pathways [90]. While downregulation of *LGALS13* in preeclampsia and other pregnancy disorders is thought to contribute to aberrant uteroplacental blood flow [41, 91], lower expression in rhesus placenta may underlie differences in the extent and depth of EVT invasion, or represent an alternative mechanism for uterine vessel expansion. Thus, our results suggest that recently evolved highly expressed human placental genes may contribute to the increased risk of aberrant placentation and preeclampsia [1]; however, further investigation of the evolutionary and functional requirements of these genes is required to confirm this notion.

Ideally, equivalent gestational ages should be used to compare DE in placentas between primates, but access to early third trimester samples (~80% gestation) from healthy human pregnancies was not possible due to obvious ethical constraints. Further, since rhesus monkeys are known to consume the placenta immediately after birth, both in captivity and in the wild, placentas from time-mated breeding pregnancies are typically collected before term to prevent the risk of losing precious third trimester rhesus placental samples. Due to these limitations, it was possible that some of the gene expression differences we detected between the human and rhesus were due to GA rather than species-specific differences. However, examination of a set of previously defined “GA-specific” placentally expressed genes [45] demonstrated that only a single GA-specific gene (*BAALC*) was differentially expressed in our analysis. Moreover, the seeming unequal distribution of male and female samples within the rhesus RNA-seq data suggested that it was possible that some of the gene expression differences we detected were sex-specific rather than species-specific differences. However, only five SDE (*ZFY*, *RPS4Y1*, *KDM5D*, *DDX3Y*, *CCK*) overlapped with DEGs identified from our cross-species analysis. Taken together, this suggests that GA- and sex-related changes accounted for a small percentage of the DEGs in our cross-species analysis, substantiating that the DE differences detected between human and rhesus placenta were largely species-specific.

Although there are previous reports of primary rhesus first and third trimester trophoblast collections [47–49], the procedure for their isolation is laborious and the cells have a finite lifespan once in culture. Immortalization of isolated primary rhesus trophoblast cells could help overcome these limitations, but such a cell line does not currently exist. In this study, we generated several TERT-immortalized cell lines from freshly isolated primary rhesus placental cells and demonstrated the robustness of the lentiviral-based TERT-immortalization approach. However, contamination with non-trophoblast stromal cells occurred in the majority of primary trophoblast isolations. Thus, additional efforts to reduce contaminating non-trophoblast cells during primary cell isolation, such as additional immunopurification steps or FACS sorting, should be implemented to increase the success rate of future attempts. In total, six TERT-immortalized rhesus placental cell lines were generated; however, only two of the lines (iRP-D26 and iRP-D28A) consisted of highly pure mononuclear trophoblast cells devoid of large-scale CNVs. These results are consistent with previous studies that revealed few karyotypic differences in TERT-immortalized cells compared to SV40-immortalized cells [92]. Nevertheless, genome duplication may still occur in TERT-immortalized trophoblast cells over time with continued passaging [7], suggesting that the genome integrity of the cell lines be routinely monitored.

In spite of broad transcriptomic similarities across human and rhesus placenta/trophoblast samples, distinct HPG expression was observed between the two species, indicating that not all human placental markers are conserved in rhesus. Transcriptomic differences between immortalized and primary rhesus trophoblasts likely reflect TERT-induced gene expression differences, changes acquired with extended culture, an enrichment of specific rhesus trophoblast cell subtypes, or the stage of differentiation captured in the immortalized cell lines. We suspect that the increase of HSV1-associated genes in iRP-D26 and iRP-D28A compared to RPTs was the result of TERT-immortalization, since HSV1 infection increases TERT activity [63]. However, the upregulation of EVT markers and extracellular matrix organization related genes is likely due to an enrichment of EVT-like cells in the immortalized cell lines compared to primary trophoblast samples. Thus, even though we intended to isolate and immortalize CTBs, it is possible that residual cell column EVTs were still attached to the villous tissue and carried over during primary trophoblast isolation or that TERT-immortalization drove the cells towards a more EVT-like phenotype during culture. We note that the same isolation procedure used here resulted in the generation of other immortalized human first trimester EVT cell lines that retained characteristics of their in vivo counterparts [4, 93, 94].

Recent single-cell RNA-seq studies of human first trimester placenta identified the presence of several different EVT and CTB subtypes [46], as well as cells at different stages of EVT/STB differentiation within primary trophoblast isolates [64]. It is well-established that several different EVT subtypes exist in human [95], and based on IHC staining and the localization of EVTs in non-human primates, there is evidence that similar EVT subpopulations also exist in rhesus [96]. Thus, the differences we detected within and between the immortalized trophoblasts and RPTs may reflect two distinct subtypes or unique stages of EVT differentiation (e.g., interstitial, endovascular, cell column) normally present in first trimester rhesus placenta. We speculate that since the iRP-D26 cell line is sensitive to forskolin treatment, it may represent an EVT subpopulation that gives rise to placental bed giant cells, while the iRP-D28A cell line represents one of the other invasive EVT subpopulations. Integration of rhesus placenta single-cell RNA-seq data would help classify the cell lines and elucidate whether these transcriptomic differences represent natural variation among rhesus trophoblast populations or are simply a byproduct of immortalization and continued culture; however, no such dataset currently exists.

While human CG (hCG) levels remain relatively high throughout human pregnancy, rhesus mCG secretion and serum levels peak at gestational day 25 and rapidly decrease to baseline levels ~10 days later [97]. Since the immortalized trophoblast cell lines were generated from rhesus placental tissue near the peak of mCG secretion, and both hCG and mCG are predominantly secreted from the STB during pregnancy, we interpreted the lack of mCG secretion by the immortalized rhesus trophoblast lines as further support for an EVT-like phenotype. It should be noted that there are previous reports of CG expression in human EVTs [98–100], and while a similar rhesus study has not been conducted in vivo, the recent derivation of rhesus trophoblast stem cells indicates that EVT-like cells do secrete CG in vitro [52]. Nonetheless, iRP-D26 and iRP-D28A showed overall transcriptomic similarity to primary rhesus trophoblasts and retained key expression and functional characteristics of invasive EVTs, highlighting the suitability of these lines for future in vitro functional investigations. Specifically, these cell lines could be used for overexpression and/or knockdown studies of the genes identified as differentially expressed in human placentas here or in other reports since primary cells, trophoblast or otherwise, are notoriously difficult to transfect. In particular, we envision assessing the function of genes shown to be upregulated in human placentas and associated with preeclampsia to determine the effects on trophoblast invasion and signaling, as well as provide insight into the mechanisms underlying the disproportionate incidence of pregnancy-related disease between humans and non-human primates.

## Conclusions

In conclusion, our comparative analysis between human and rhesus bulk placenta showed that while a majority of HPGs are similarly expressed between the two species, certain genes are differentially expressed between human and rhesus placenta. These results suggest that rhesus is a suitable surrogate for most investigations of human placentation; however, notable molecular differences related to EVT function and preeclampsia should be considered and further interrogated in future investigations. Moreover, we generated immortalized rhesus trophoblast cell lines that represent a useful tool for primate placental investigations, especially for in vitro experiments that interrogate the putative function of genes identified in this study. Transcriptomic comparison and functional assessment of these cell lines suggest that they retain attributes of rhesus primary first trimester EVTs. Collectively, the results of this study (1) provide a comprehensive list of genes differentially expressed between human and rhesus placenta that informs the translatability of primate placental investigations, (2) help delineate the underlying molecular basis of increased EVT invasion and heightened susceptibility to preeclampsia and other pregnancy-related diseases in human, and (3) offer a reliable source of first trimester rhesus trophoblasts for current and future in vitro studies of early primate placentation.

## Methods

### Tissues and cell lines

Deidentified human term placental samples were collected by and acquired through the Labor and Delivery Unit at the Oregon Health and Science University Hospital and deposited into a repository under a protocol approved by the Institutional Review Board with informed consent from the patients. A total of five different human placentas from healthy cesarean section term births, ranging from 38.9 to 41.3 gestational weeks, were used for RNA-seq library generation (Additional file 2). All rhesus monkey (*Macaque mulatta*) tissues were collected in compliance with the guidelines established by the Animal Welfare Act for housing and care of laboratory animals and conducted per the Institutional Animal Care and Use Committee (IACUC protocols #0514 and #0580) at the Oregon National Primate Research Center (ONPRC). All rhesus placentas were collected from time-mated breeding pregnancies delivered via cesarean section. Two frozen rhesus third trimester placental samples, collected at 140 and 141 gestational days, were used for RNA isolation and qRT-PCR validation of DE analysis results. Six fresh rhesus placentas were used for primary rhesus trophoblast TERT-immortalization, including two term placentas (D141, D149) and four first trimester placentas (D26, D28A, D28B, D50). An additional first trimester rhesus placenta (D50) was used for primary trophoblast culture and RNA-seq analysis; these cells were not included in TERT-immortalization experiments. For all samples, the placentas were separated from the fetus and amniotic sac, collected in cold sterile saline and immediately processed for isolation of primary trophoblasts. The primary female and male rhesus macaque skin fibroblasts cell lines, Fb.XX (AG08312) and Fb.XY (AG08305), were acquired through Coriell Institute. Frozen stocks of the highly pure rhesus first trimester trophoblast cell lines, iRP-D26 and iRP-D28A, were generated at various passage numbers and can be made available to researchers upon request. While earlier passages were used for the initial characterization (passages 8–26), later passages were subjected to functional analyses, including Matrigel invasion, forskolin treatment, and hormone/growth factor secretion (passage 19-32) (Additional file 16).

### RNA isolation and purification

Frozen placental samples were ground into a powder using liquid nitrogen-cooled mortar and pestle then directly added to TRIzol reagent (Thermo Fisher #15596026); for cell lines media was removed and TRIzol reagent was added directly to the tissue culture dish. RNA was isolated from TRIzol reagent, treated with Turbo DNAse (Thermo Fisher #AM1907), and purified using RNA Clean and Concentrator-5 spin columns (Zymo #R1013) according to manufacturer's instructions.

### RNA-seq library preparation and sequencing

NEBNext® Ultra II Directional RNA Library Prep Kit for Illumina and NEBNext rRNA Depletion Kit (NEB, Ipswich, MA) was used to generate RNA-seq libraries from purified RNA following the manufacturer’s instructions. Libraries were quantified with the Qubit High Sensitivity dsDNA Assay (Invitrogen, Carlsbad, CA), and size distribution was assessed with a 2100 Bioanalyzer High Sensitivity DNA Analysis Kit (Agilent). Multiplexed bulk human placental libraries were sequenced on the NextSeq500 platform using 150 cycle single-end protocol generating a total of 36.9 to 70.9 million 101bp reads per sample. Multiplexed rhesus trophoblast cell lines were sequenced on the NextSeq500 platform using 100 cycle single-end protocol generating a total of 57.8 to 68.5 million 75bp reads per sample.

### Human and rhesus orthologous gene annotations

Human protein-coding gene annotations (GRCh38.99) including associated rhesus orthologous gene annotations (Mmul10.99) were downloaded from ENSEMBL BioMart [101, 102]. Gene annotations used for DE analysis were filtered to include only human protein-coding genes with “high-confidence” “one2one” rhesus orthologous genes, producing a final set of 15,787 human gene annotations and associated 15,787 rhesus orthologs (Additional file 3). A total of 13,471 orthologs genes passed the minimum DEseq2 default expression threshold for differential expression statistical analysis.

### Differential expression (DE) analysis

For human and rhesus cross-species DE analysis, raw fastq files were trimmed of low-quality and adapter sequences using Trimmomatic [103] and mapped to both the human (GCh38) and rhesus (Mmul10) reference genomes using Bowtie2 [104] with a very sensitive parameter. Resulting BAM files were filtered to remove low quality and multi-mapped reads (MAPQ ≥10) using samtools [105] view -q 10. Raw read counts for GRCh38.99 human gene annotations were generated from GRCh38 mapped data, while raw read counts for Mmul10.99 rhesus gene annotation were generated from Mmul10 mapped data, using featureCounts [106]—primary and filtered to include gene annotations described above. Gene counts were normalized and DEGs (padj<0.05 & Log2FC>|2|) were identified using default setting of DEseq2 [107]. DE analysis was performed with human mapped data (DE-GRCh38) and with rhesus mapped data (DE-Mmul10). A gene was considered differentially expressed only if it was identified as significantly (padj<0.05) upregulated or downregulated (|L2FC|>2) by both DE-GRCh38 and DE-Mmul10 analyses. The DE analysis was repeated a total of three times, with three independent sets of human placental RNA-seq data (Additional file 1: Fig. S2A-C). The first DE analysis included the five human placental RNA-seq samples generated by our group (DE#1), the second DE analysis included six publicly available human placental RNA-seq datasets (DE#2), and the third DE analysis included two publicly available human placental RNA-seq datasets (DE#3); all three DE analyses included the same four publicly available rhesus placental RNA-seq datasets. The final set of DEGs consisted only of genes determined to be significantly upregulated or downregulated by all three DE analyses.

The DE analysis between male and female human placental samples was performed as described above with the exception that trimmed reads were mapped exclusively to the human reference genome. Additionally, the DE analysis between immortalized trophoblast and RPT cells was performed as described above with the following exceptions: (1) trimmed reads were mapped exclusively to the rhesus reference genome, (2) differential expression was analyzed for all 21,575 protein-coding rhesus gene annotations (ENSEMBL v98), and (3) a gene was identified as significantly differentially expressed if padj<0.05 and Log2FC>|1|.

PCA and heatmap visualizations were generated using DEseq2 variance stabilizing transformation (VST) normalized human gene count data. PCA analysis was performed with VST-normalized expression data from all genes included in DE analysis. Morpheus webtool (https://software.broadinstitute.org/morpheus) was used to generate heatmaps and perform hierarchical clustering (metric: one minus Pearson correlation, linkage method: complete). Statistical over-representation analysis of the human and rhesus upregulated gene lists was performed using g:Profiler webtool [108]. Custom background gene lists containing the final 15,787 orthologs described earlier and 21,575 rhesus protein-coding genes were used for human/rhesus upregulated and iRP over-representation tests, respectively. Query DEG lists were tested for over-representation of several functional genes sets, including the default g:Profiler biological pathway (KEGG, Reactome, and WikiPathways) gene sets; Human Protein Atlas (HPA) trophoblast subtypes gene sets (https://www.proteinatlas.org/humanproteome/celltype); “Rare_Diseases_GeneRIF_Gene_Lists” and “Jensen_DISEASES” human disease gene sets extracted from Enrichr webtool [109, 110] (https://maayanlab.cloud/Enrichr/).

### qRT-PCR

Primers were carefully designed to amplify both human and rhesus sequences of all genes examined, with noted exceptions (Additional file 17). Purified RNA was reverse transcribed into complementary DNA (cDNA) using SuperScript VILO cDNA Synthesis Kit (Thermo Fisher #11754050). Samples were prepared for high-throughput qRT-PCR using 96.96 gene expression dynamic array (Fluidigm BioMark) following manufacturer's protocol “Fast gene expression analysis using Evagreen.” Briefly, preamplification of cDNA was performed using a 500-nM pooled primer mix, unincorporated primers were removed with exonuclease I treatment, and diluted 5-fold before samples and detectors were loaded and run on a 96.96 array with the following thermocycler settings: 70°C for 40 min, 60°C for 30 s, 95°C for 1 min, 40 cycles of 95°C 15 s, 59.5 °C for 15 s, and 72 °C for 15 s. Two no template control (NTC) samples and four technical replicates of each reaction were included. The analysis was performed using qbase+ software, with GAPDH, HPRT1, and TBP serving as the reference genes used for normalization. Statistical significance was determined using two-sided unpaired t test with alpha of 0.05. Mean calibrated normalized relative quantities (CNRQ) were exported from qbase+, Log_2_ transformed, then used as input for heatmap generation with Morpheus web tool.

### Transcriptomic comparison of human, rhesus, and mouse placenta

Human gene annotations (GRCh38.101) including associated rhesus (Mmul10.101) and mouse (GRCm38.101) orthologous gene annotations were downloaded from Ensembl BioMart [101, 102]. Gene annotations were filtered to include only human protein-coding genes with “one2one” rhesus and mouse orthologous genes. The orthologs were further filtered to exclude ribosomal genes and genes from chromosomes X, Y, and MT, producing a final set of 14,054 orthologous human, rhesus, and mouse 1:1:1 gene annotations. The method used for transcriptomic comparison of three species is largely based off a previously described approach [111]. For this, we used RNA-seq data generated in-house from human (*n*=5) and publicly available rhesus (*n*=4) and mouse (*n*=4) placenta RNA-seq data (Additional file 2). Raw fastq files were trimmed of low-quality and adapter sequences as described above. Transcripts per million (TPM) gene expression values were calculated using RSEM v1.3.1 [112] with reference genome and gene annotation downloaded from Ensembl version 101 [101, 102] (Additional files 4, 5, 6). For this, trimmed data were mapped to respective genomes/gene annotations (RSEM index) using Bowtie2 [104] with the following parameters --sensitive --dpad 0 --gbar 99999999 --mp 1,1 --np 1 --score-min L,0,−0.1. DEseq2 was used for normalization and differential expression calling, with TPM values as inputs. One plus Log_2_ normalized TPM values were used for heatmap generation and hierarchical clustering using Morpheus webtool. DE results were filtered to include only genes with |L2FC|>2 and padj<0.05.

### Human placental marker gene (HPG) set analysis

HPGs were defined by combining previously identified placenta “tissue enriched” and “group enriched” genes from The Human Protein Atlas [31]. A total of 190 human placental markers were extracted from this database, including 91 “placenta-enriched” genes having at least four-fold higher mRNA level in the placenta compared to any other tissue, and 99 “placenta group-enriched” genes having at least four-fold higher average mRNA level in a group of 2–5 tissues compared to any other tissue [31]. Human placental marker genes lacking an ENSEMBL-defined “one2one” or “high-confidence” rhesus orthologous gene could not reliably be compared and were excluded from our DE analysis.

### Rhesus primary trophoblast cell isolations

Primary trophoblast cells were isolated from rhesus placentas using protocols adapted from previously described methods for human first trimester tissue [4, 113, 114] and human term tissue [115]. All rhesus placentas were obtained immediately after cesarean section delivery, and procedures were performed in a biosafety cabinet using ice-cold and sterile solutions unless otherwise noted. Placental tissue was transferred to a Petri dish and covered with sterile saline, and the villous tissue was dissected from the decidua and chorionic plate using scissors and forceps; decidua and fetal membranes were discarded. To remove any contaminating blood, the villous tissue was washed until clear with several changes of sterile saline then crudely minced using scissors.

For first trimester placentas, villous tissue was transferred to a 50-mL tube containing warmed 0.25% trypsin solution and incubated at 37 °C for 10 min, mixed by inverting every 2–3 min. The tissue was allowed to settle at bottom of the tube for 5 min before the supernatant was discarded, and the tissue was washed with three changes of 1X phosphate-buffered saline without Ca_2_+ and Mg_2_+ (PBS--) (Caisson Labs #PBL05). To release the CTB, the tissue was transferred to a fresh Petri dish containing warmed 0.25% trypsin 0.2mg/mL DNAse I solution and a scalpel or glass slide was used to thoroughly scrape the villi. The surrounding trypsin solution containing desired CTBs was collected through a 70-μm cell strainer into 50 -mL tubes containing 5 -mL fetal bovine serum (FBS) (Fisher #16-140-063). Cells were centrifuged at 300 g for 10 min and resuspended in Hanks Balanced Salt Solution (HBSS). This suspension was carefully layered over an equal volume of Lymphocyte Separation Media (Corning #25-072-CI) in a 15-mL conical tube, and centrifuging the gradient at 400g for 15min with the break off. While the red blood cells collected as a pellet at the bottom of the tube, the interface between the HBSS and LSM, containing the trophoblast cells, was carefully removed using a transfer pipet. The cells were pelleted then resuspended in cell culture media (CCM): DMEM high-glucose glutaMAX (Fisher, #10566-016), 10% FBS, 100 U/mL Pen-Strep (Fisher #15-140-148).

For term placentas, villous tissue was digested for 30 min shaking at 37 °C with 0.25% trypsin and 0.2mg/mL DNAse I. The supernatant was reserved and the digest was repeated two additional times. The three digests were combined and centrifuged. Pellets were resuspended in DMEM and re-pelleted. Cells were carefully layered over a preformed Percoll gradient layered at 60, 55, 50, 45, 35, 30, and 25%, prior to centrifugation at 2800 rpm for 30 min without brake. The CTB cells between 35% and 55% were collected, counted and resuspended in CCM. For both first trimester and term placentas, cells were centrifuged at 300g for 10 min, and the cell pellet was resuspended to 10^8^ cells/mL in 1X Nanobead buffer (BioLegend #480017). Contaminating immune cells were depleted using anti-CD45 Magnetic Nanobeads (BioLegend #488028) following the manufacturer's instructions. Purified trophoblast cells were resuspended in complete trophoblast media (CTM): MEM – Earle’s with D-Val (Caisson Labs #MEL12), 10% normal human serum (Gemini Bio #100-110), 100-U/mL Pen-Strep, 1-mM Sodium Pyruvate (Fisher #11-360-070), and 0.1M HEPES (Fisher #15-630-106); and grown on enhanced tissue culture dishes (Corning Primaria, #C353802) in a humidified 37 °C environment with 5% CO_2_. Primary rhesus trophoblasts included in the RNA-seq (D50B) were harvested after nanobead immuno-purification and an additional CTM wash step.

### TERT-immortalization

Primary rhesus trophoblasts and rhesus skin fibroblast cells were immortalized using Alstem’s TERT-immortalization kit (Alstem #CILV02) following the manufacturer's instructions. In brief, following the isolation of primary cells or 24 h after thawing of skin fibroblasts, the cells were plated at a density of 1.5 × 10^5^ cells/well in a 6-well plate and transduced the following day. Each well received 1 mL of media containing 4 μL recombinant TERT lentivirus and 500x TransPlus reagent (Alstem # V020). After 16 h, the media were replaced with fresh culture media and the cells were allowed to recover for 48 h before beginning puromycin selection (Santa Cruz Biotechnology #SC-108071). Cells were treated with 800ng/mL puromycin for a total of 72 h. The surviving cells were propagated and represent the TERT-immortalized cell lines established and characterized throughout these studies. Mock transductions using the same transduction conditions without lentivirus added to media were included throughout puromycin selection to ensure depletion of non-transduced cells.

### Cell culture

All cell lines were grown in a humidified 37 °C environment with 5% CO_2_. Cell culture media was changed every 2 days, and cells were enzymatically passaged using TrypLE (Gibco). Primary and TERT-immortalized trophoblast cell lines were cultured in CTM, while fibroblast samples were cultured in CCM. iRP-D26 and iRP-D28A cell lines were passaged at a density of 30,000 cells/cm^2^, while all other lines were passaged at a density of 15,000 cells/cm^2^.

### DNA sequencing and chromosome copy number calling

Cells were dissociated using TryplE, pelleted, and resuspended in PBS^--^ containing 0.05% trypsin-EDTA (Thermo Fisher Scientific). A stereomicroscope was used to isolate, wash, and collect cells into individual sterile PCR tubes. Immediately after collection, PCR tubes containing single-cells (*n*=4), five-cells (*n*=1), and ten-cells (*n*=1) were flash-frozen on dry ice, and stored at −80°C until library preparation. Individual samples underwent DNA extraction, whole-genome amplification, library pooling, and DNA sequencing were performed as previously described [55]. Multiplexed libraries were loaded at 1.6pM and sequenced on the NextSeq 500 platform using the 75 cycle single-end protocol. The resulting sequencing data was filtered, trimmed, and mapped to the rhesus reference genome (Mmul8) as previously described [55]. Chromosome copy number calling and plots were generated using Ginkgo [116]. The proportion of Chr Y reads was determined for each sample by dividing the number of reads mapped to Chr Y by the total number of mapped reads. The relative proportion of Chr Y reads was identified by normalizing samples to the known male sample (iRFb-XY), and samples with a mean relative proportion ≥ 0.50 were identified as male.

### Metaphase spread chromosome counts

Cells were treated with a 0.015-ug/mL colcemid overnight (~12 h) to induce metaphase arrest. Cells were dissociated using TryplE, pelleted, and resuspended in a warm hypotonic solution (0.06 M KCl, 5% FBS) for 15 min before being fixed with 3:1 methanol:acetic acid. Slides were made and baked at 95C for 20 min, cooled, trypsinized for 45 s, and stained with Wright’s stain. At least 20 metaphase spreads were analyzed and brightfield images captured using 100X objective on a Nikon microscope and counted using FIJI software.

### IHC staining

Paraffin sections were deparaffinized and rehydrated through xylene and a graded alcohol series, then washed for 5 min in running tap water. Antigen unmasking was performed using sodium citrate (pH 6.0) buffer in a pressure cooker for 20 min and washed in three changes of PBS. An endogenous enzyme block was performed by incubating sections in 0.3% hydrogen peroxide for 10 min and washed in three changes of PBS. Nonspecific proteins were blocked by incubating sections in 5% horse serum for 30 min. Primary antibodies were diluted as described for IF staining, and the tissue was incubated in primary antibody dilutions for 2 h at room temperature. Mouse IgG H+L (Vector Labs, BA-2000) and rabbit IgG H+L (Vector Labs, BA-1100) biotinylated secondary antibody dilutions were prepared at 1:250 in PBS + 1% BSA. The tissue sections were incubated in the secondary antibody dilution for 1 h at room temperature, then washed in three changes of PBS. VECTASTAIN Elite ABC HRP Kit (Vector Laboratories, PK-6100), and ImmPACT DAB Peroxidase HRP substrate (Vector Labs, SK-4105) were used according to manufacturer's instructions. Nuclei were counterstained with hematoxylin (Electron Microscopy Sciences, 26043-05) and imaged using a brightfield microscope.

### IF staining

The cell culture media was removed from the cells, fixed with ice-cold methanol for 15 min at −20°C, then washed in three changes of PBS. Nonspecific proteins were blocked by incubating cells in 5% donkey serum for 30 min. Anti-KRT7 (mouse monoclonal, Dako, M7018), anti-CDH1 rabbit monoclonal (Cell Signaling, 3195S), anti-VIM rabbit monoclonal (Cell Signaling, 5741T), anti-PTPRC rabbit monoclonal (Cell Signaling, 13917S), and anti-SDC1 mouse monoclonal (Miltenyi Biotec, 130-119-927) antibodies were diluted in PBS + 1% BSA (KRT7 1:250, CDH1 1:250, VIM 1:250, CD45 1:250) and incubated for 2 h at room temperature. The cells were then washed in three changes of PBS, before incubating in secondary antibody dilutions for 1 h at room temperature. Alexa Fluor 488 and 594 (Life Technologies) secondary antibody dilutions were prepared at 1:1000 in PBS + 1% BSA. Cells were counterstained with DAPI and washed with three changes of PBS before imaging. Images of cells were captured using the 20X objective on an epifluorescence microscope or a Leica SP5 AOBS multi-spectral confocal microscope and processed using FIJI software.

### Forskolin fusion assay

The cell lines were passaged onto 6-well plates and treated the following day with either media containing 25-uM forskolin or DMSO. After 48 h, two wells from each condition were processed for RNA isolation and a single well was processed for IF staining as described above. Four technical replicates were included in subsequent qRT-PCR analysis, and five micrographs of immunostained cells were captured per condition.

### Monkey chorionic gonadotropin (mCG) concentrations by radioimmunoassay (RIA)

mCG IRA was performed on media collected after 48 h in culture from iRP-D26, iRP-D28A, and iRP-D28B cell lines treated with 25uM forskolin (*n*=3) or DMSO (*n*=3). Highly purified human CG (LER hCG) for radioiodination and reference standard was obtained from Dr. Leo Reichert, Tucker Endocrine Research Institute; Tucker, GA [117]. Each ampule of human CG contained 50 micrograms. An ampule of hCG was re-dissolved in 50 ml of phosphate-buffered saline (PBS) so that the concentration of hCG was 1 mg/ml. After being completely dissolved, the hCG was separated into aliquots of 5 mg/5 ml for radioiodination. For reference standards, 24.95 ml of 1% BSA-PBS was added to one hCG ampoule, resulting in a concentration of 200 ng/ml which was divided into 0.5 ml per aliquot and stored at −80°C. Ovine antiserum H-26 has been established in RIA and used extensively for measuring monkey CG concentrations in the blood, urine, and cells [118]. While the antiserum was generated against ovine luteinizing hormone (LH), it was targeted toward monkey CG, and, for a very long time, has been the only antibody available to a few laboratories for measuring monkey CG. H-26 antiserum was diluted to 1:2000 for use as the primary antibody in our laboratory. For precipitation, the anti-rabbit gamma globulin, NIH #1, was diluted to 1:50 for use in the mCG RIA. Assay tubes were centrifuged at 3000 rpm for 30 min in a Beckman J-6 refrigerated centrifuge. Before the assay, LER hCG was radioiodinated with Iodine-125 (I-125) (Perkin-Elmer, Billerica, MA) for use as a trace. Briefly, 1.0 mCi of fresh I-125 was mixed with 5 μg of LER hCG for 1 min under oxidative conditions. The reaction was stopped by adding reductive solution, and the I-125-labeled hCG was separated from free I-125 by column chromatography (using Bio-Gel P60, 200-300 wet mesh, from Bio-Rad, Hercules, CA). The fractions containing proteins were tested with antibody within 24 h, and the specific and non-specific bindings were assessed. All samples for mCG determinations were assayed at original concentration. Overall, the characteristics of the standard curves indicated that the assays were well executed. Of the 12 standard points in serial dilutions of hCG from 10 ng/ml to 0.0049 ng/ml, 10 maintained dose-response between 5 and 95% binding. The highest point (10 ng/ml) and lowest point (0.0049 ng/ml) were outside the 5% confidence limit at the upper and lower end of the standard curve, respectively. However, these standard points did not affect the calculation of samples as the sample values were based on their specific bindings. The sensitivity of the assay was estimated to be 0.1 ng/ml (at about 90% binding).

### Trans-well migration and Matrigel invasion assay

24-well trans-well inserts with 8uM pores (Falcon #353097) were coated with a 1.2-mg/mL Matrigel (Corning #354234), following the manufacturer’s instructions. Migration and invasion assays were performed by culturing cells at 37°C for 48 h on uncoated (*n*=3) and Matrigel-coated (*n*=3) trans-well inserts, respectively. Assays were carried out under both normoxia and hypoxia (~1% O_2_) conditions. A total of 25,000 cells in 250-μL serum-free media were added to each insert, and 650 μL of complete trophoblast media was added to each surrounding insert. At 48 h, the cells remaining on the topside of the insert were wiped away with sterile cotton swap before the insert was fixed with 4% paraformaldehyde and the nuclei were stained with DAPI. The 10X objective of a Nikon Eclipse epifluorescence microscope was used to capture five micrographs of the bottom side of each insert, and the number of cells/nuclei were counted automatically using FIJI software with default threshold and measure functions. For each insert, the mean average cell counts across the five micrographs were used to calculate the “average cell counts per insert” reported. For each cell line, the mean average cell counts across all three uncoated inserts were used as the denominator for calculating the percent invasion (coated/uncoated) represented. A two-sided t test with alpha of 0.05 was used to determine significance between conditions.

### IGF2 secretion assay

Culture media samples were collected from cell lines cultured for 48 h (*n*=2 for: iRFb, iRP-D26, iRP-D28A, iRP-D28B; *n*=4 for rhesus serum pool), then centrifuged for 5 min at 300g to remove cellular debris before IGF-2 secretion analysis. Secreted IGF-2 concentrations were determined by ELISA following the manufacturer’s instructions (R&D Systems DG-100, Minneapolis, MN) in the Endocrine Technologies Core (ETC) at ONPRC. The assay range was 12.5–800 pg/mL. Intra-assay variation for an in-house monkey serum pool was 4.0%. All samples were quantified in a single assay, and no inter-assay variation was determined. The assay was validated for use in monkey samples by the ETC prior to the analysis of samples. This validation included analysis of a dilution series to test for assay specificity as well as a spike and dilution analysis to test for analyte recovery and matrix effects.

## Supplementary Information

**Additional file 1.** Supplemental figures.

**Additional file 2.** Samples and sequencing details.

**Additional file 3.** DE results of human versus rhesus bulk placenta (all 15,787 genes).

**Additional file 4.** Human TPM-normalized gene expression values.

**Additional file 5.** Rhesus TPM-normalized gene expression values.

**Additional file 6.** Mouse TPM-normalized gene expression values.

**Additional file 7.** TPM-based human versus mouse placenta DEGs.

**Additional file 8.** TPM-based human versus rhesus placenta DEGs.

**Additional file 9.** DE results of human versus rhesus bulk placenta (190 HPGs).

**Additional file 10.** ORA results of human and rhesus upregulated DEGs.

**Additional file 11.** Significant DEGs in male versus female human bulk placenta.

**Additional file 12.** DE results of iRP-D26 versus RPT (all 21,578 genes).

**Additional file 13.** DE results of iRP-D28A versus RPT (all 21,578 genes).

**Additional file 14.** ORA results of iRP-D26 upregulated and downregulated DEGs.

**Additional file 15.** ORA results of iRP-D28A upregulated and downregulated DEGs.

**Additional file 16.** Immortalized cell line passage details.

**Additional file 17.** Primer details.

## Data Availability

All data generated or analyzed during this study are included in this published article and its supplementary information files. The RNA-seq and DNA-seq raw sequencing data generated in this study have been submitted to the NCBI Sequencing Read Archive (SRA) under BioProject accession number PRJNA649979 (https://www.ncbi.nlm.nih.gov/bioproject/PRJNA649979). Additional publicly available RNA-seq data from human, rhesus, and mouse were all downloaded from NCBI SRA using SRA Toolkit (http://ncbi.github.io/sra-tools/): human placenta (DE#2): SRR3096525, SRR3096545, SRR3096594, SRR3096612, SRR3096624, SRR3096625 [27]; human placenta (DE#3): SRR4370049, SRR4370050 [28]; HPT: SRR2397323, SRR2397324, SRR2397332, SRR2397333, SRR2397341, SRR2397342, SRR6443608, SRR6443609, SRR6443611, SRR6443612 [57, 58]; human PBMC: SRR3389246, SRR3390437, SRR3390461, SRR3390473 [58]; human BeWo: SRR6443610, SRR6443613, SRR6443614, SRR6443615, SRR6443616, SRR9118949, SRR9118950 [61, 62]; rhesus placenta: SRR5058999, SRR5059000, SRR7659021, SRR7659022 [26]; rhesus PBMC: SRR2467156, SRR2467157, SRR2467159, SRR2467160 [59]; mouse placenta: SRR649373, SRR649374, SRR943344, SRR943345 [29].

## Abbreviations

CCM: Cell culture media
cDNA: Complementary DNA
CNRQ: Calibrated normalized relative quantity
CNV: Copy number variation
CTB: Cytotrophoblast
CTM: Complete trophoblast media
DE: Differential expression
DEGs: Differentially expressed genes
EVT: Extravillous trophoblast
HPG: Human placental marker gene
HPT: Human primary trophoblast
IF: Immunofluorescent
IHC: Immunohistochemistry
iRFb: TERT-immortalized rhesus fibroblast cell line
iRP: TERT-immortalized rhesus placental cell line
L2FC: Log_2_ fold change
LSM: Lymphocyte separation media
MAPQ: Mapping quality
not DE: Not differentially expressed
NTC: No template control
PAC: Puromycin resistance
PBMC: Peripheral blood mononuclear cells
PCA: Principle component analysis
qRT-PCR: Quantitative reverse transcription PCR
RPT: Rhesus primary trophoblasts
STB: Syncytiotrophoblast
TERT: Telomerase
