# Supplementary material for: Transcriptomic analysis of primate placentas and novel rhesus trophoblast cell lines informs investigations of human placentation
Source: BMC Biol. 2021 Jun 21;19:127. doi: 10.1186/s12915-021-01056-7 (PMC8218487; doi:10.1186/s12915-021-01056-7)
Supplement: Supplementary file 1 — Additional file 1. Supplemental figures. [file 12915_2021_1056_MOESM1_ESM.docx]

Transcriptomic analysis of primate placentas and novel rhesus trophoblast cell lines informs investigations of human placentation

*Jimi L. Rosenkrantz (rosenkrj@ohsu.edu)^1,2^, Jessica E. Gaffney (jessgaffney@gmail.com)^2^, Victoria HJ. Roberts (robertsv@ohsu.edu)^2^, Lucia Carbone (carbone@ohsu.edu)^1,3-5*^ and Shawn L. Chavez (chavesh@ohsu.edu)^1,2,6,7*^*

*^1^Department of Molecular and Medical Genetics, Oregon Health and Science University, Portland, OR 97239; ^2^Division of Reproductive and Developmental Sciences, Oregon National Primate Research Center, Beaverton, OR 97006; ^3^Division of Genetics, Oregon National Primate Research Center, Beaverton, OR 97006; ^4^Department of Medicine, Knight Cardiovascular Institute, Oregon Health and Science University, Portland, OR 97239; ^5^Department of Medical Informatics and Clinical Epidemiology, Oregon Health and Science University, Portland, OR 97239; ^6^Department of Obstetrics and Gynecology, Oregon Health and Science University School of Medicine, Portland, OR 97239; ^7^Department of Biomedical Engineering, Oregon Health and Science University School of Medicine, Portland, OR 97239*

*To whom correspondence should be addressed:

Shawn L. Chavez, Ph.D.

505 NW 185^th^ Avenue

Beaverton, OR 97006

email: chavesh@ohsu.edu

phone: 503-346-5423

Lucia Carbone, Ph.D.

3303 SW Bond Avenue

Portland, OR 97239

email: carbone@ohsu.edu

phone: 503-494-7342

**Supplemental Figures**


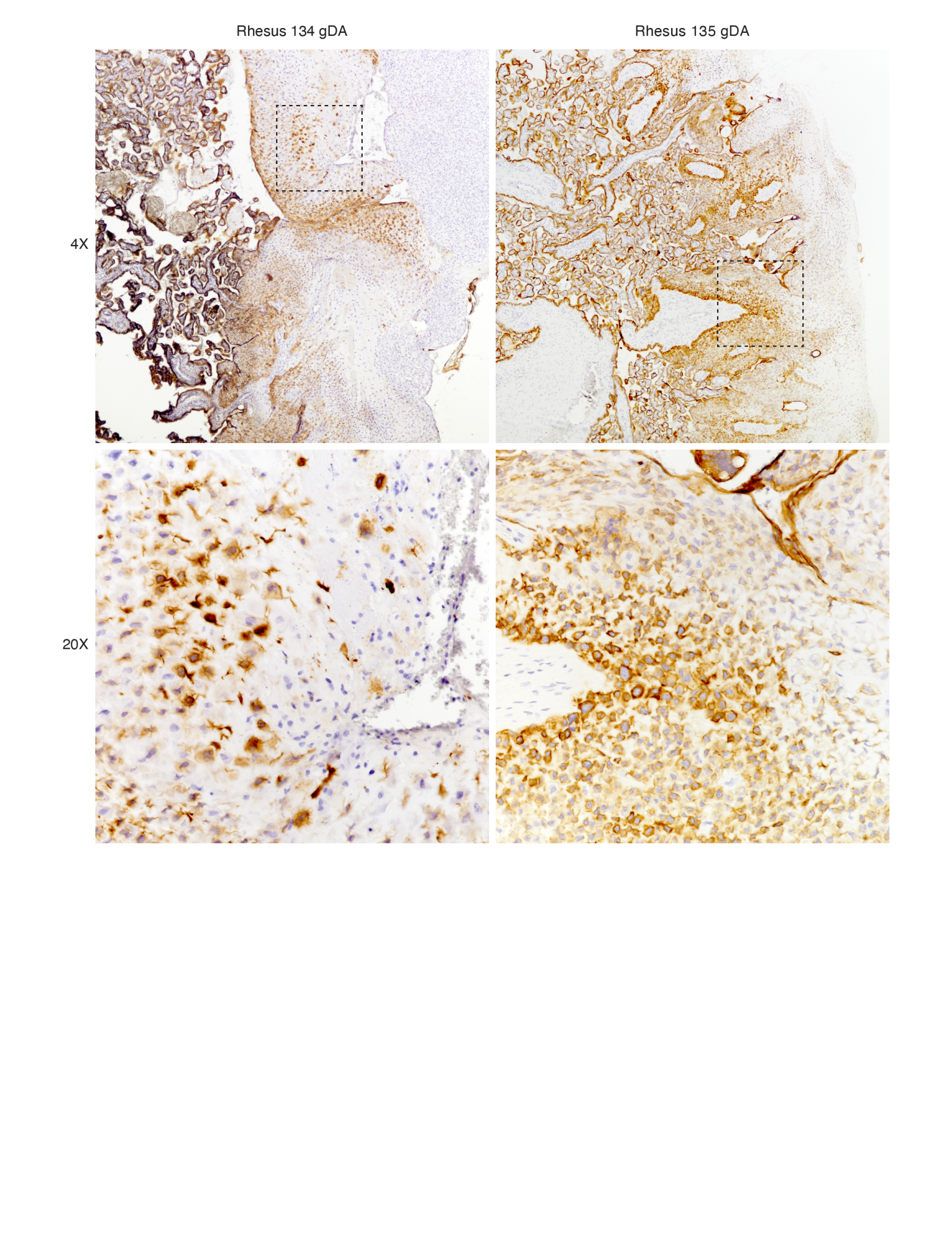


**Supplemental Figure S1. Rhesus placenta IHC.** Rhesus placental tissue at ~80% gestation stained for KRT7 (brown). Tissue from gestational day 134 (left) and 135 (right), imaged using 4X (top) and 20X objective (bottom). Dashed boxes denote regions examined at higher magnification.

**
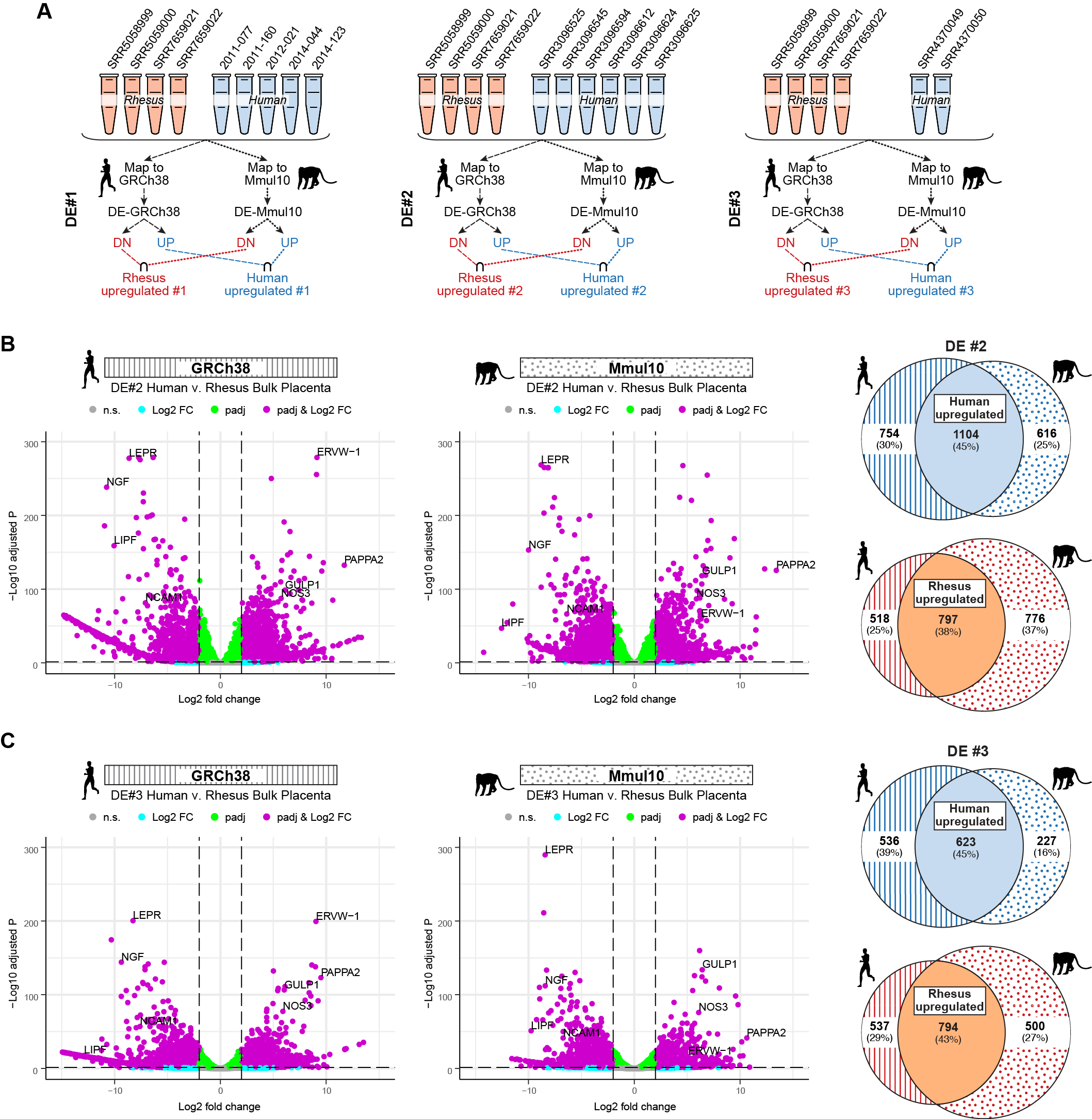
**

**Supplemental Figure S2. Human versus rhesus placenta differential expression.** (A-C) Schematic of DE analysis (A) DE#1 (B) DE#2 (C) DE#3. (D,E,G,H) Volcano plots showing gene expression fold differences between human and rhesus term placental tissue (n=8) from (D,E) DE#2 (n=6) and (G,H) DE#3 (n=2) using data mapped to (D,G) human genome and gene annotations (DE-GRCh38), and data mapped to (E,H) rhesus genome and gene annotations (DE-Mmul10). Dashed lines denote DE significance (padj<0.05) and fold change (|L2FC|>2) thresholds; genes passing significance threshold (green), passing L2FC threshold (cyan), passing both (magenta), or none (grey). (F,I) Venn-diagram depicting intersection of DE-GRCh38 (stripes) and DE-Mmul10 (spotted) results to identify intermediate human upregulated (light blue) and rhesus upregulated (light red) genes sets from (F) DE#2 and (I) DE#3.


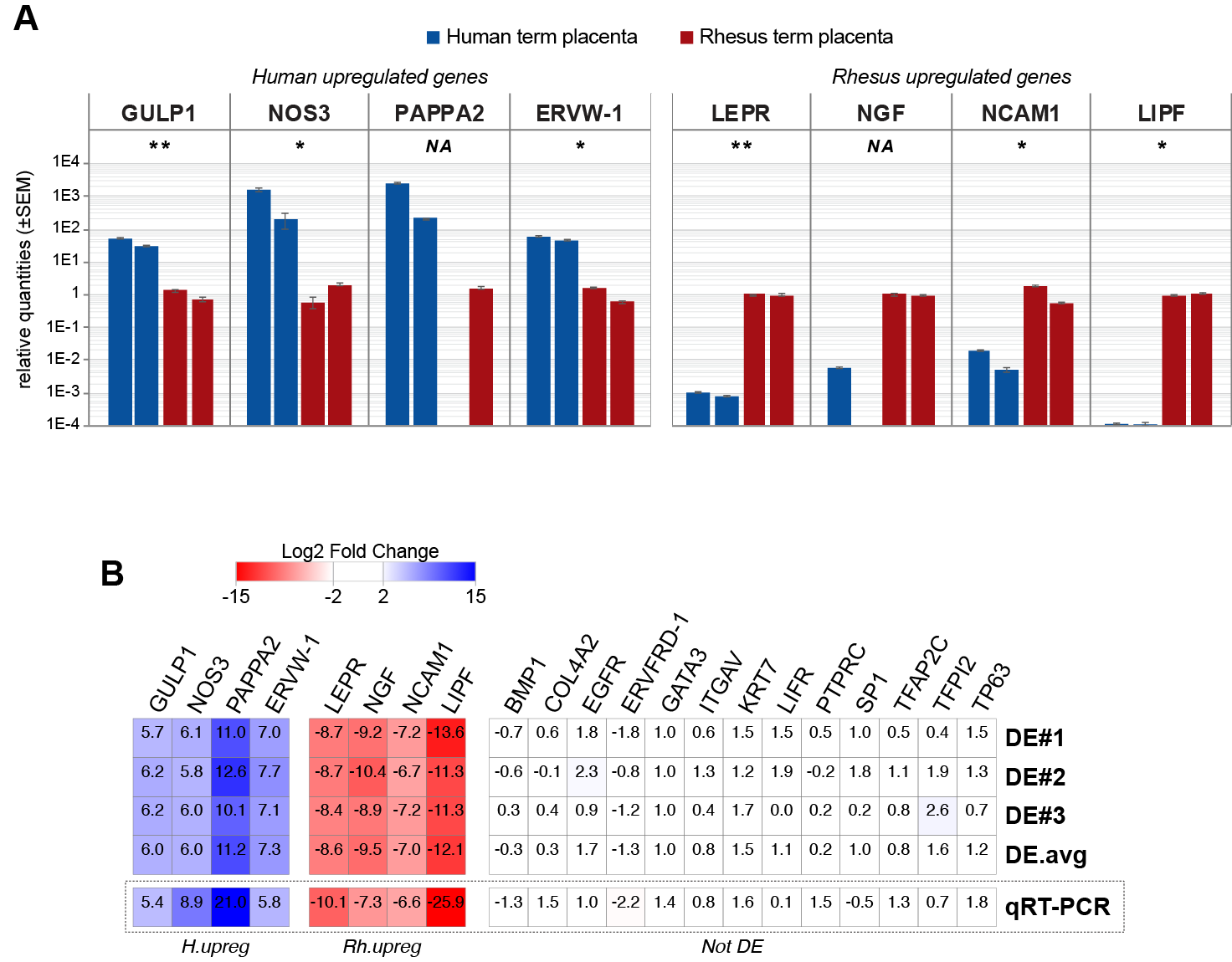


**Supplemental Figure S3. qRT-PCR validation of DEGs identified from DE analysis.** (A) qRT-PCR relative gene expression quantities (mean ± SEM) of DEGs (n=8) in human (blue) and rhesus (red) bulk placental samples; all samples were normalized to *GAPDH*, *HPRT1*, and *TBP*, and scaled to rhesus bulk placental samples. Technical replicates (n=4) for the PCR reaction and biological replicates (n=3) for each species group were included. Statistically significant differences between human and rhesus groups were identified using two-sided unpaired t-test with alpha of 0.05 (*p<0.05, **p<0.01). (B) Heatmap depicting human versus rhesus log2 fold-change values determined via qRT-PCR and the RNA-seq based DE analyses.

**
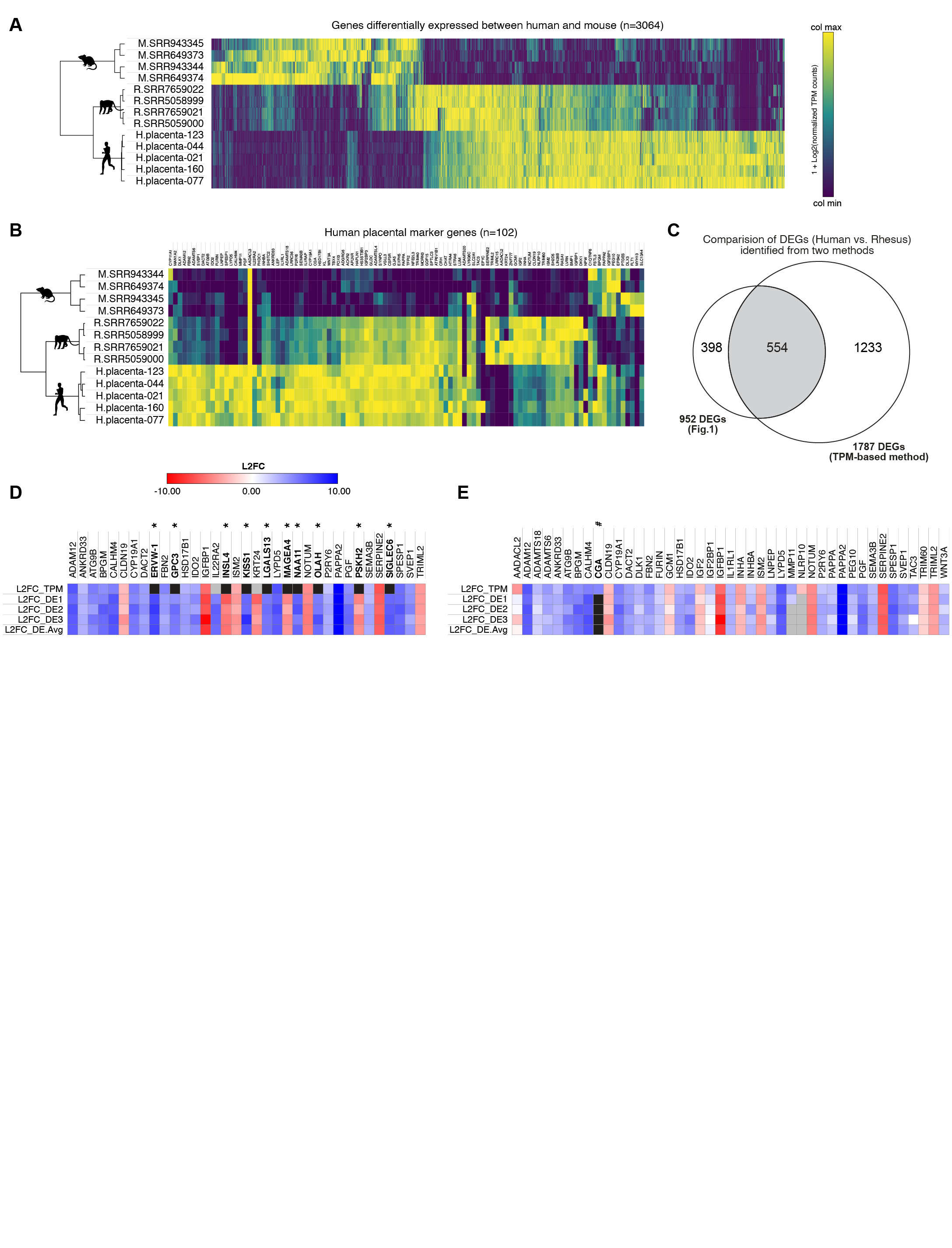
Supplemental Figure S4. TPM-based transcriptomic comparison of human, rhesus and mouse placenta.** (A) Heatmap depicting expression levels [Log2(1+ TPM)] of genes differentially expressed between human (n=6) and mouse (n=4) placenta and (B) HPGs. Hierarchal clustering shows rhesus samples cluster more closely to human than mouse samples for both gene sets **(Additional file 7)**, highlighting molecular similarities between human and rhesus (n=4) placental samples. (C) Venn-diagram depicting the intersection of human versus rhesus DEGs (**Additional file 8**) identified from our primary DE approach (left) and TPM-based DE strategy (right). (D) Heatmap comparing Log2 fold change of differentially expressed HPGs identified from the primary DE approach between human and rhesus, and (E) TPM-based DE strategy; grey boxes denote NA values; black boxes denote excluded genes; *=genes excluded from TPM-based analysis due to lack of mouse orthologue; #=genes excluded from primary DE analysis due to low-confidence rhesus orthologue. ­­


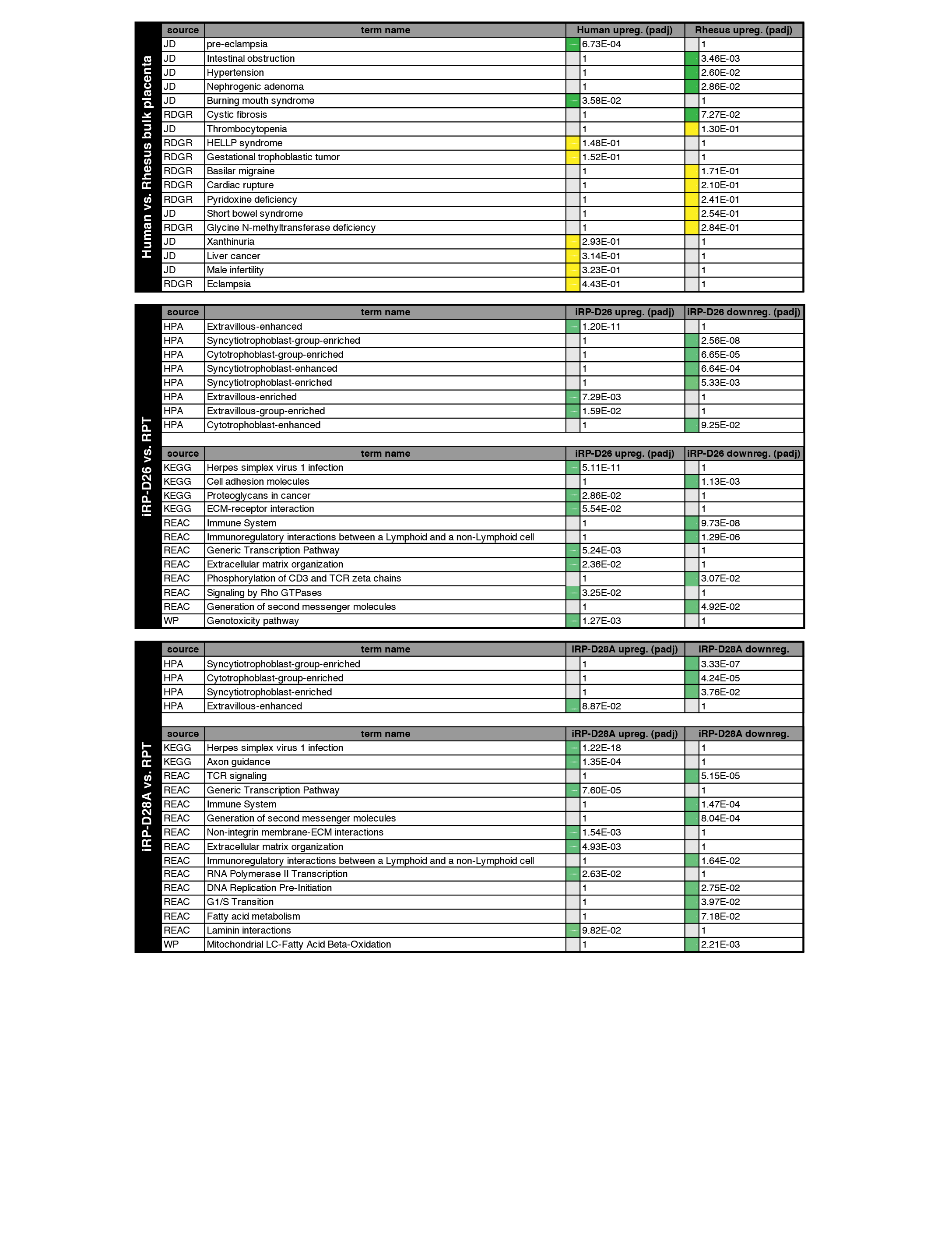
**Supplemental Figure S5. Functional enrichment analysis.** Over-representation analysis **(Additional file 10)** of human-upregulated and rhesus-upregulated DEG sets (top), iRP-D26 upregulated and downregulated DEG sets (middle), and iRP-D28A upregulated and downregulated DEG sets (bottom); functional terms with padj<0.1 (green), padj<0.5 (yellow); JD=Jensen_DISEASES, RDGR=Rare_Diseases_GeneRIF_Gene_Lists, HPA=Human Protein Atlas, REAC=Reactome, WP=WikiPathways.


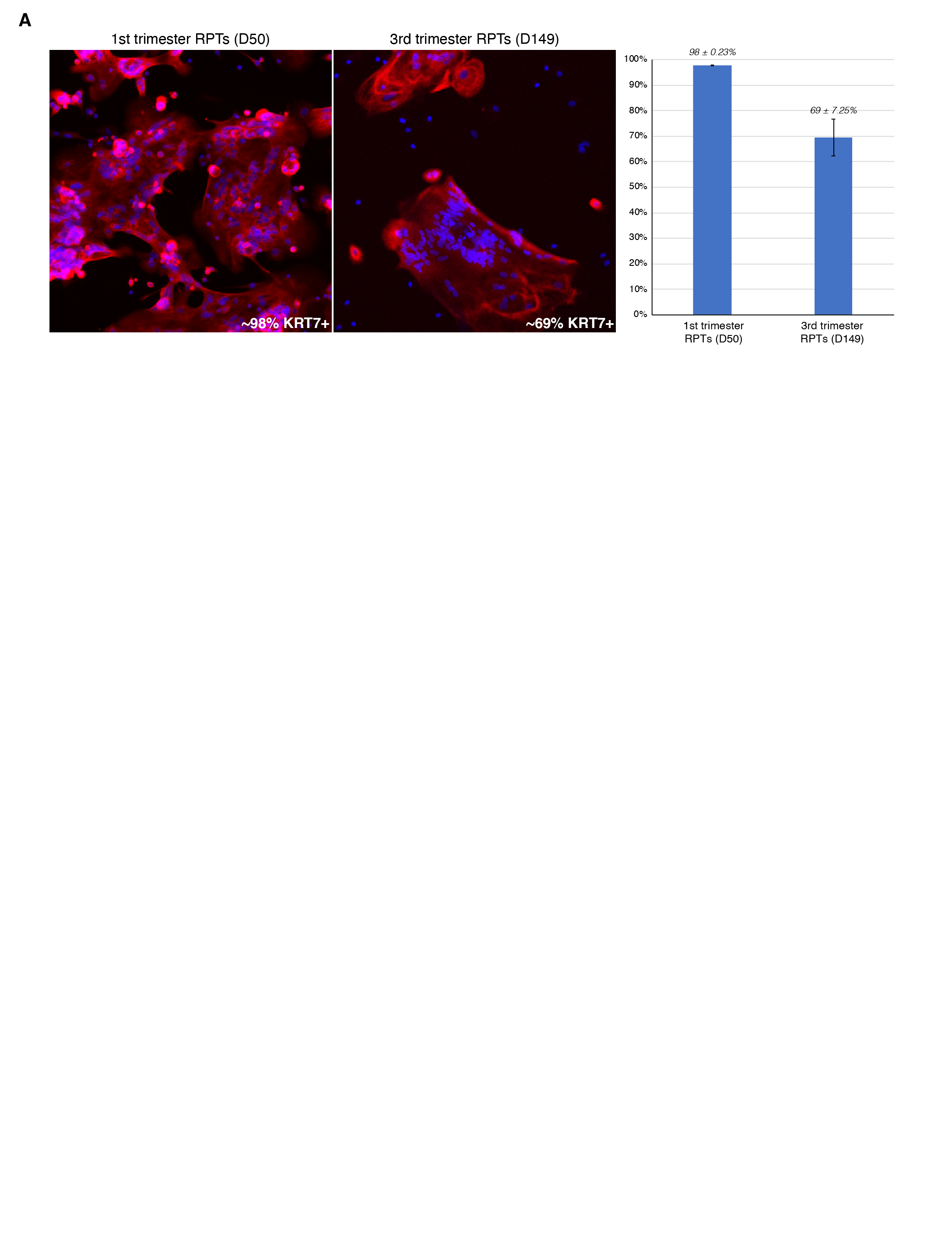


**Supplemental Figure S6. Purity of primary rhesus trophoblast cell isolations.** KRT7 IF staining of primary rhesus trophoblast cells isolated from D50 first trimester (left) and D149 third trimester (middle) rhesus placenta. Bar graph depicts average percent KRT7 positive cells calculated (n=5) across immunostained micrographs from 1^st^ (n=1) and 3^rd^ (n=1) trimester primary cell cultures.

**
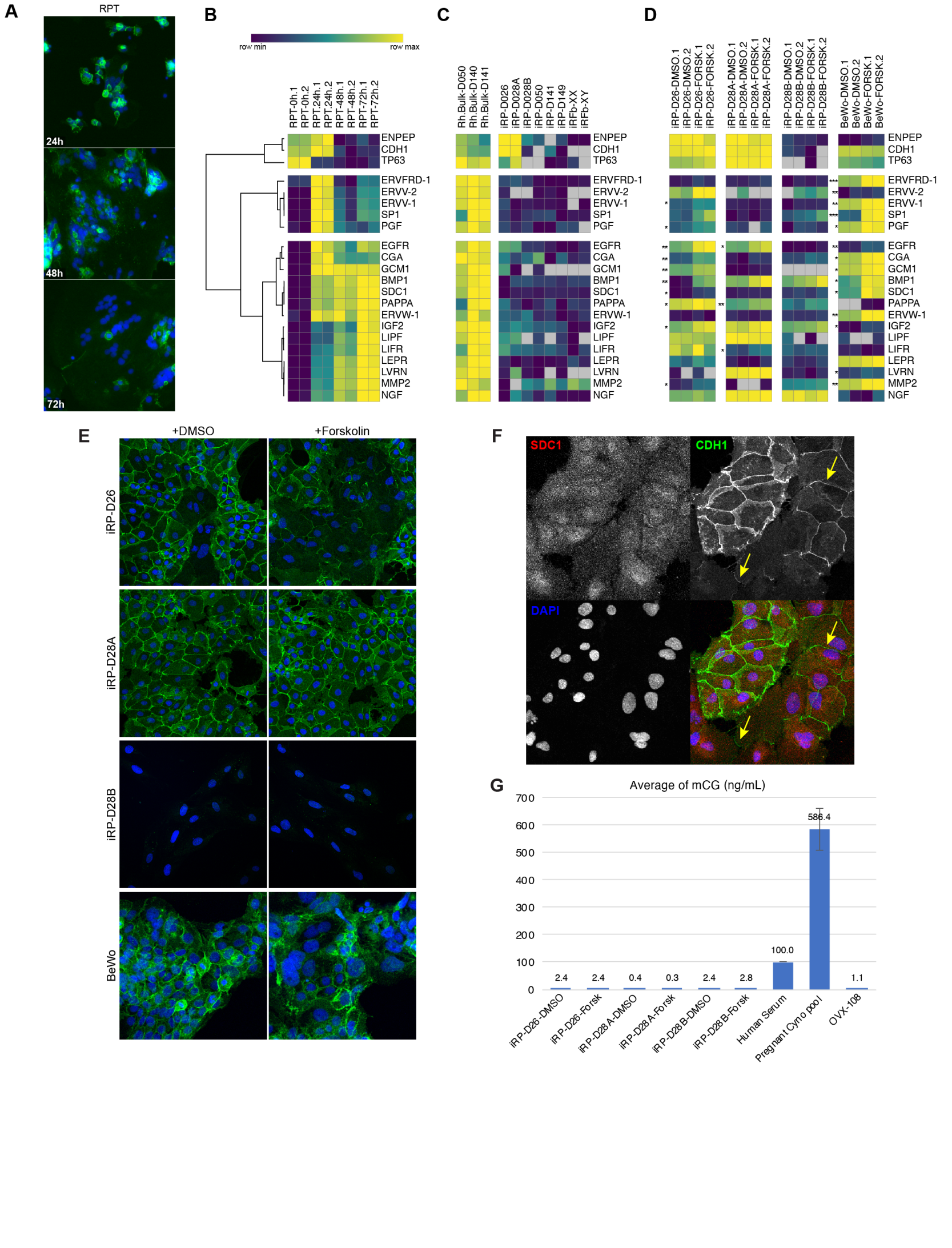
**

**Supplemental Figure S7. Functional characterization of primary and immortalized rhesus trophoblast cells.** (A) CDH1 IF staining of RPT cells after 24 h, 48 h, and 72 h in culture, showing spontaneous fusion/syncytialization of RPTs (B-D) Heatmaps of qRT-PCR RNA expression levels. Color scale depicts minimum (purple) and maximum (yellow) Log2 relative gene expression values compared across (B) RPT cells after 24 h, 48 h, and 72 h in culture (C) bulk rhesus placenta, iRP, and iRFb cell lines; (D) iRP and BeWo cell lines treated with DMSO (n=2) or Forskolin (n=2) for 48 h. Statistically significant differences between DMSO and forskolin treatment groups were identified using two-sided unpaired t-test with alpha of 0.05 (*p<0.05, **p<0.01, ***p <0.001). (E) CDH1 IF staining of iRP and BeWo cell lines treated with DMSO or Forskolin for 48 h (F) IF staining confocal maximum intensity projected microphotographs of a region showing reduced CDH1 staining in iRP-D26 forskolin-treated culture. Faint CDH1-positive plasma membrane surrounding most nuclei is observed (arrows); CDH1 (green), SDC1 (red), DAPI (blue). (G) Bar graph of mCG secretion concentrations detected via radioimmunoassay (RIA) in cell culture media from iRP-D26, iRP-D28A, iRP-D28B, human serum, and in serum from pregnant (n=3) and ovariectomized (OVX) cynomolgus macaques (n=1).
